# Digital Phenotyping via Passive Network Traffic Monitoring: Prospective Observational Study in University Students

**DOI:** 10.2196/84618

**Published:** 2026-04-27

**Authors:** Rameen Mahmood, Annabelle David, Donghan Hu, Nabil Alshurafa, Lou M Haux, Josiah Hester, Andrew Kiselica, Shinan Liu, Chenxi Qiu, Chao-Yi Wu, Zachary Beattie, Jeffrey Kaye, Danny Yuxing Huang

**Affiliations:** 1 Department of Electrical and Computer Engineering, Tandon School of Engineering, New York University, 370 Jay St, Brooklyn, NY, 11201, United States, 1 (646) 997-0500; 2 Center for Urban Science + Progress (CUSP), NYU Tandon School of Engineering, New York University, Brooklyn, NY, United States; 3 Department of Preventive Medicine, Northwestern University, Chicago, IL, United States; 4 Department of Computer Science, Northwestern University, Chicago, IL, United States; 5 Max Planck Institute for Human Development, Berlin, Germany; 6 College of Computing, Center for Advancing Responsible Computing, Georgia Institute of Technology, Atlanta, GA, United States; 7 Institute of Gerontology, Department of Health Policy and Management, University of Georgia, Athens, GA, United States; 8 Department of Data and Systems Engineering, University of Hong Kong, Hong Kong, China (Hong Kong); 9 Department of Neurology, Beth Israel Deaconess Medical Center, Harvard Medical School, Boston, MA, United States; 10 Department of Medicine, Beth Israel Deaconess Medical Center, Harvard Medical School, Boston, MA, United States; 11 Broad Institute of MIT and Harvard, Cambridge, MA, United States; 12 Neurology, Massachusetts General Hospital, Harvard Medical School, Charlestown, MA, United States; 13 Oregon Alzheimer’s Disease Research Center & Center for Aging & Technology (ORCATECH), Oregon Health & Science University, Portland, OR, United States

**Keywords:** passive sensing, digital phenotyping, encrypted network traffic, remote monitoring, digital behavior

## Abstract

**Background:**

Digital behaviors such as sleep, social interactions, and productivity reflect how individuals structure their daily lives. Among university students, online activity patterns mirror academic schedules, social rhythms, and lifestyle habits, with disruptions linked to sleep, stress, and well-being. Existing approaches—including wearables, apps, and surveys—depend on self-report or active participation, limiting long-term adherence. Passive sensing of network traffic offers a scalable alternative for the unobtrusive capture of smartphone usage patterns that preserves privacy.

**Objective:**

This study evaluated the degree to which encrypted smartphone network traffic, collected via a standard virtual private network (VPN), can capture patterns of digital behavior. We assessed feasibility (sustained data capture) and acceptability (usability, burden, and privacy perceptions) and examined how traffic-derived features reveal aspects of digital behavior—including timing, intensity, and regularity—relevant to health and daily functioning.

**Methods:**

We conducted a 2-week prospective observational study at New York University. Participants installed the WireGuard VPN client on personal smartphones, enabling passive capture of encrypted network traffic. Feasibility was assessed using a mixed methods approach combining quantitative measures of user retention and data coverage with qualitative analysis of semistructured exit interviews. Acceptability was evaluated using the System Usability Scale, NASA Task Load Index, and qualitative interview analysis. Exploratory analyses visualized traffic-derived features in relation to digital activity patterns.

**Results:**

Thirty-eight students consented, of whom 29 (76.3%) contributed valid network traffic data and formed the analytic cohort. Within this cohort, 93% of participants (27/29; Wilson 95% CI 78%‐98%) contributed at least 5 days of monitoring, corresponding to 71% retention relative to all consented participants (27/38; Wilson 95% CI 55%‐83%). The mean data coverage within the analytic cohort (n=24) was 74.1% (SD 19.3%; median 77.1%, IQR 63.6%-90.0%; bootstrap 95% CI 66.3%‐81.4%). These participants contributed an average of 311.6 (∼13 d, SD 3.5) hours of monitored traffic, ranging from 121 to 496 hours. Acceptability outcomes were evaluated among participants completing the exit survey and interview. Usability ratings were high (System Usability Scale score: mean 78, SD 14.96), and perceived workload was low (NASA Task Load Index scores were minimal). Participants described the system as easy to install, unobtrusive, and generally trustworthy, although some reported temporarily disabling the VPN during activities they considered private. No inferential statistical tests were conducted; analyses were descriptive. Exploratory analyses indicated that traffic-derived features reflected daily digital activity rhythms and revealed distinctive lifestyle patterns, including gaming and irregular late-night food delivery use.

**Conclusions:**

VPN-based monitoring of encrypted smartphone traffic was feasible and acceptable, enabling sustained passive data collection with minimal burden. This approach shows promise as a scalable, device-agnostic method for digital phenotyping that captures fine-grained behavioral rhythms while preserving privacy. With broader validation, this technique could expand the toolkit for studying health and well-being in everyday life.

## Introduction

### Background

Many changes relevant to health and well-being manifest as gradual shifts in the timing and regularity of everyday activities, rather than as isolated events captured at a single point in time [1-4]. Consequently, commonly used episodic assessments—such as surveys, retrospective self-reports, or infrequent evaluations—often miss subtle but meaningful changes in daily behavior that precede or accompany shifts in health and functioning [5]. Prior work has consistently linked disruptions in behavioral rhythms—such as sleep disturbance and irregular daily routines—to psychiatric and cognitive conditions. This highlights the potential for these everyday behavioral patterns to be early indicators of changes in well-being [6-42]. Notably, many such behavioral alterations are detectable well before clinical diagnosis, underscoring the potential value of passively collected digital behaviors as low-burden, noninvasive markers of emerging health risk [2,3,43-47].

Digital behaviors—defined by how people interact with devices and digital platforms—offer a practical window into changes in daily routines, social engagement, and rest-activity organizations as they unfold over time [48-58]. Among university students, digital behaviors mirror academic, social, and lifestyle demands, and irregularities have been tied to stress and poorer health [59-66]. Although much of the existing evidence comes from clinical contexts, it reflects a broader principle: health-relevant behavioral change is often expressed in the timing, regularity, and structure of everyday activities. When captured passively through digital traces, these behavioral rhythms can serve as low-burden markers of changes in well-being, even in nonclinical populations.

Yet, capturing such behaviors in real-world contexts remains constrained by the limitations of current measurement approaches. For example, polysomnography is the gold standard for quantifying sleep architecture and fragmentation, but it is expensive, invasive, and typically limited to single-night laboratory assessments [67,68]. Wearable and sensor-based systems offer the possibility of extending monitoring into daily life [69-72], although their adoption is often limited by the psychological burden of continuous device use, adherence challenges, battery consumption, restricted coverage, and behavioral changes they impose [39,68,73-81]. These limitations have motivated researchers to seek more naturalistic, low-burden sources of behavioral data, most notably smartphones [82-84].

Deeply embedded in the daily routines of young adults, smartphones generate continuous digital traces that reflect a wide range of everyday rhythms, including social interaction, online engagement, mobility, and rest-activity cycles [82,85]. Prior work has shown that messaging activity patterns can reveal diurnal rhythms [86,87], while periods without device use can approximate disengagement, such as sleep or downtime [88,89]. While such signals cannot capture fine-grained quality measures, they provide scalable proxies for broader temporal patterns of behavior. In our study, we leverage passive digital traces—captured through encrypted network traffic—from everyday smartphone use to estimate key behavioral regularities, such as activity onset and offset, usage intensity, and daily rhythm stability, without relying on self-reports, surveys, or other active input from users.

### Prior Work

Translating digital traces into meaningful behavioral indicators requires methods that are scalable, minimally burdensome, and privacy-preserving. Achieving this balance remains challenging under current mobile operating system constraints. iOS and Android restrict continuous background monitoring by third-party sensing apps, while built-in analytics tools such as Apple’s Screen Time and Android’s Digital Wellbeing provide only coarse-grained summaries (eg, daily or weekly app totals) without fine-grained temporal context [90-94]. More intrusive approaches—including rooting, jailbreaking, or continuous screen capture—impose substantial participant burden and raise privacy concerns, limiting their suitability for long-term, real-world deployment [88,95,96].

Despite these constraints, prior work has demonstrated that digital behaviors carry meaningful behavioral signals. In young adults, late-night and irregular device use has been associated with disrupted sleep and depressive symptoms [97-99], while broader patterns of messaging, app engagement, and disengagement have been linked to mood, social well-being, and cognitive function [89,100-104]. These findings underscore the promise of digital behavior as a scalable proxy for well-being, even as existing sensing approaches remain technically and ethically constrained.

Current approaches reflect a trade-off between burden and precision. Self-report instruments are easy to deploy but suffer from recall errors, social desirability bias, and participant fatigue when repeated frequently [5,105-107]. Higher-resolution approaches, including custom tracking apps, offer greater accuracy but demand intrusive permissions and elevated privileges [75,79,108,109]. Mobile sensing platforms such as the Effortless Assessment of Risk States and Beiwe have been widely deployed in behavioral health research [110-116], yet they depend on operating system (OS)–level application programming interfaces (APIs; eg, iOS Screen Time), participant-mediated configuration (eg, whitelisting), sustained device permissions, which can substantially limit data yield: in a large-scale deployment of the Effortless Assessment of Risk States involving 4754 adolescents, fewer than half (n=1463, 30.8%) contributed analyzable data [117]. Screenomics [118] provides fine-grained, content-level capture via frequent screenshots [119], but at the cost of substantial intrusiveness, privacy concerns, and limited scalability for long-term use [117,120,121].

These limitations highlight the need for methods that balance resolution, scalability, and privacy while reducing participant burden. Network-level approaches offer one promising direction, as encrypted traffic metadata can capture the *timing*, *regularity*, and *intensity* of digital engagement—behavioral dimensions that prior work has linked to sleep, mood, and cognitive functioning [122-125]—without accessing content or requiring elevated permissions. Prior studies have shown that such metadata can reveal coarse app categories [126] and device-level behaviors [127-129], although evaluations have typically been limited in scope or deployment setting. Our study extends this line of work by evaluating a VPN-based, metadata-only sensing framework in a real-world deployment and assessing the ability of network traffic–derived features to recover health-relevant behavioral rhythms under constraints of feasibility and acceptability relevant to longitudinal use. A broader contextual comparison with prior mobile sensing approaches is provided in the *Relation to Prior Work* section.

### Goal of This Study

The goal of this study was to evaluate the feasibility and acceptability of a VPN-based, network-level sensing approach and to assess the ability of network traffic–derived features to capture daily behavioral patterns relevant to health and well-being. Specifically, we characterized network traffic–derived features as markers of daily behavioral patterns—including activity onset and offset, usage regularity, and the stability of daily routines—in a real-world university deployment.

Unlike many existing tools, this approach requires no app whitelisting or custom keyboards, imposes negligible battery overhead, and avoids intrusive permissions, making it suitable for longitudinal deployments in everyday settings. The network traffic data we collected mirror what internet service providers or mobile carriers routinely observe, but with stronger safeguards: all traffic is anonymized and never shared with third parties [130]. By enabling continuous, low-burden observation of everyday digital behavior, this approach provides a foundation for studying how patterns of screen use, sleep timing, and daily behavioral rhythms relate to health and well-being over time.

Building on this rationale, we structured the study around the following aims:

- Aim 1 (feasibility): to evaluate the feasibility of continuous, passive capture of encrypted network traffic on participants’ devices for supporting app usage inference
- Aim 2 (acceptability): to evaluate participants’ perceptions of the monitoring method, with emphasis on usability, privacy, and burden
- Exploratory aim: to explore the ability of network traffic–derived features to capture health-relevant daily behavioral patterns, including sleep-wake cycles, usage regularity, and activity rhythms

Together, these aims position our approach as a scalable, privacy-preserving foundation for longitudinal research on digital behavioral rhythms with implications for health outcomes. Given the single-site university setting and the high baseline digital literacy of the study cohort, the feasibility and acceptability observed here should be interpreted as an upper bound relative to what may be achievable in more diverse or older populations.

## Methods

### Study Tools

We conducted a 2-week observational study with university students at New York University (NYU) to evaluate the feasibility and acceptability of passive digital phenotyping using VPN-based smartphone network traffic. Participants installed the WireGuard VPN app [131] on their personal smartphones, enabling continuous collection of encrypted network traffic metadata. Feasibility and acceptability were assessed using a mixed methods approach combining quantitative measures with standardized usability instruments—the System Usability Scale (SUS) [132] and the NASA Task Load Index (NASA-TLX) [117]—and semistructured exit interviews. In addition to feasibility and acceptability, we conducted exploratory analyses to investigate traffic-derived features as markers of broad daily behavioral patterns.

### Participants and Recruitment

Participants were recruited through academic advisors across multiple NYU schools and departments who distributed study invitations via internal mailing lists. Recruitment began in April 2025 and targeted university students who were willing to participate in a 2-week passive monitoring study. Interested students received a consent packet describing study procedures and privacy considerations before enrolling.

**Figure 1.**
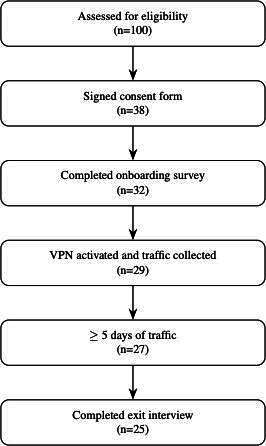
CONSORT (Consolidated Standards of Reporting Trials)–style participant flow diagram, showing progression from initial recruitment through study completion. VPN: virtual private network.

As this was a feasibility pilot, no predetermined sample size was specified; recruitment continued on a best-effort basis within the study time frame. As shown in the CONSORT (Consolidated Standards of Reporting Trials) diagram (Figure 1), the final cohort reflects the number of participants who were successfully recruited and onboarded during the study period. Detailed demographic characteristics are reported in Multimedia Appendix 1. Additional details regarding recruitment rationale, outreach procedures, and onboarding workflow are provided in Multimedia Appendix 2.

### Study Instrument: WireGuard VPN for Passive Monitoring

A central challenge in mobile behavioral research is capturing objective measures of smartphone use without modifying the OS, requesting intrusive permissions, or imposing sustained battery and attention costs. Existing approaches—such as platform-specific monitoring apps or rooted devices—are challenging to scale, fragile to OS updates, and raise privacy concerns.

To address these limitations, we used a virtual private network (VPN) [133] to passively capture encrypted network traffic metadata. When active, the VPN routes all device-to-internet traffic through a study-managed server, allowing the observation of high-level metadata such as destination hostnames, packet counts, and byte volumes, while leaving all packet contents end-to-end encrypted. This approach is device agnostic and OS agnostic, requires no app-level permissions or whitelisting, and preserves content privacy by design.

**Figure 2.**
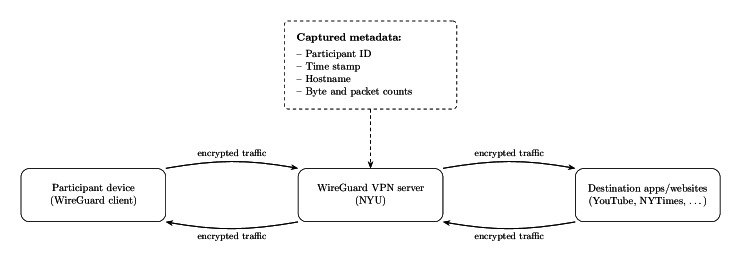
System architecture. All device-to-internet traffic is routed through a New York University (NYU)–hosted WireGuard virtual private network (VPN) server. Although packet contents remain end-to-end encrypted, the server captures metadata (eg, hostnames and traffic volumes), enabling passive behavioral analysis without accessing message content.

We implemented this approach using the WireGuard VPN protocol, selected for its cross-platform availability and robustness in continuous operation. Participants installed the official WireGuard client and connected via a personalized configuration, after which traffic metadata were passively logged in fixed time windows for analysis (Figure 2). In laboratory testing, the VPN imposed negligible battery overhead (<1% over 24 h), enabling sustained, unobtrusive monitoring.

Additional technical details regarding VPN configuration, server infrastructure, and data aggregation are provided in Multimedia Appendix 3.

### Field Study

#### Onboarding Process

After enrollment, participants completed setup through a secure web portal that generated a personalized VPN configuration, which they installed using the official WireGuard app. The installation status was verified prior to the start of data collection. Full onboarding procedures and portal design details are provided in Multimedia Appendix 4.

#### Data Collection

Once the WireGuard VPN was activated on a participant’s device, it remained active by default and routed all device-to-internet traffic through the study server unless it was explicitly disabled or interrupted (eg, by a device reboot). During active periods, the server captured encrypted traffic metadata aggregated in 10-second windows [134]. Packet contents were never collected.

This design allowed us to observe *when* and *how intensively* apps were used without access to content. For example, when a participant streamed YouTube, we could observe traffic exchanged with services such as *youtube.com* and the corresponding traffic volume, but not which specific video was viewed. The same principle applied to news sites, banking portals, and messaging apps: we captured only high-level traffic characteristics, never sensitive user content.

Participants could manually disable the VPN, and unintentional downtime could also occur (eg, following device reboot). Visibility of VPN status varied across devices: older iPhones display a persistent VPN icon in the status bar, newer iPhones with a notch, and most Android devices omit such an indicator. As a result, participants may be unaware when the VPN is inactive.

To mitigate data gaps and support adherence, the study protocol incorporated reminder emails sent every 2 to 3 days, directing participants to a study portal where they could verify connection status.

**Figure 3.**
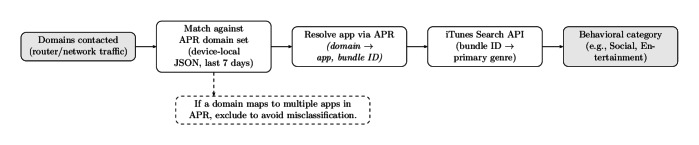
Hostname → app → category pipeline. Domains observed in network traffic are matched against Apple’s App Privacy Report (APR) to recover the generating app (via bundle ID), which is then linked to its primary genre using the iTunes Search API. This process yields higher-level behavioral categories that complement app-level mappings. API: application programming interfaces.

**Figure 4.**
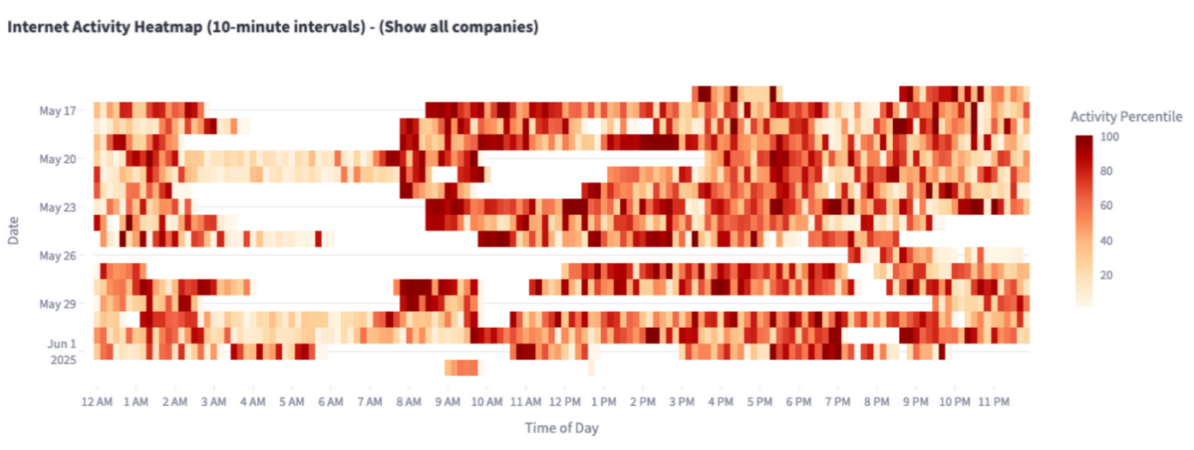
Internet activity heatmap aggregated in 10-min intervals over 24 h. Rows represent study days, and columns represent time of day; color intensity indicates the percentile of observed activity.

The portal provided real-time feedback on cumulative active study days and compensation earned (Figure D.1 in Multimedia Appendix 5), serving both as a technical safeguard against accidental disengagement and as a behavioral nudge to maintain adherence. Beyond payment tracking, the portal also provided a transparency dashboard that allowed participants to see the types of hostnames and apps being observed and to visualize their activity patterns over time (Figures 3 and 4). These views were designed to contextualize participants’ own digital behavior while reinforcing transparency regarding the scope of data collection (Figure 4).

Implementation parameters, storage formats, aggregation settings, and compensation thresholds are documented in Multimedia Appendix 5.

#### Privacy Model

To prevent linkage between digital behaviors and real-world identities, each participant created a random participant ID (PID) during onboarding; no mapping between PIDs and personal information was stored, and participants could regenerate a new PID at any time. The VPN server logs *metadata only*—PID, time stamp, destination hostname, and byte or packet counts; packet contents are never collected. Data were stored on NYU-managed infrastructure with encryption and restricted access to authorized study personnel. Before enrollment, participants received a plain-language briefing (slides, video, and live demonstration) describing the privacy model and risks and provided informed consent.

#### Exit Surveys and Interviews

At the conclusion of the study, participants were invited to complete a short exit survey. The survey collected free-text entries for the top 10 apps used during the study period, along with standardized usability assessments: the NASA-TLX and the SUS.

Participants were also invited to a semistructured follow-up interview conducted via Zoom (Zoom Video Communications, Inc. [135]) to reflect on their experience with VPN-based monitoring. Interview prompts focused on VPN usage, engagement with the study portal, perceived burden, and willingness to extend participation beyond the 2-week study period. The interview format prioritized participant-led discussion, allowing concerns and observations to surface organically. A complete set of interview questions is provided in Multimedia Appendix 6.

To preserve anonymity while enabling compensation, study completion and payment were decoupled from collected metadata. The study portal generated a completion code encoding the participant’s compensation amount without linking it to their participant identifier (PID). Participants presented this code during the interview to confirm eligibility for payment without revealing their study identity. Additional details on interview scheduling and compensation verification are described in Multimedia Appendix 7.

As an optional step, participants using iPhones could choose to share their Apple App Privacy Report (APR), an Apple-generated summary of recent app activity (described in the section *Data Analysis*). Participants were briefed on the scope and limitations of this report, which indicates app usage timing but not accessed content. Shared reports served only as a complementary reference for app usage, augmenting the network metadata collected through the VPN.

### Data Analysis

To evaluate feasibility, acceptability, and the potential of passive traffic data to capture everyday activity patterns, we combined qualitative insights from participant interviews with quantitative analysis of network traffic metadata.

#### Qualitative Analysis: Interviews

Two authors independently reviewed interview transcripts [136] and conducted an inductive thematic analysis [137-140], generating an initial set of 127 codes. Through iterative axial coding, the code set was refined by merging overlapping categories, revising definitions, and removing redundancies. Themes were developed through consensus-based synthesis to capture recurring patterns in participants’ experiences related to feasibility, acceptability, and trust. This approach was selected to allow themes to emerge directly from participants’ accounts while maintaining analytic transparency and reproducibility. The complete qualitative codebook, including first-cycle codes and axial groupings, is provided in Multimedia Appendix 8.

#### Quantitative Analysis: Feasibility

We evaluated feasibility across 2 domains, that is, user retention and data coverage, following conventions in digital phenotyping and mobile sensing research.

#### User Retention

User retention was defined as the proportion of participants who contributed at least 5 days of traffic out of all participants who contributed any traffic. This threshold ensured that estimates were based on individuals with a meaningful amount of monitoring.

#### Data Coverage

Data coverage was defined as the proportion of 60-minute intervals (hours) within each participant’s monitoring window (from their first observed active hour to their last) that contained valid VPN traffic. An hour was considered “active” if at least one byte of data was observed during that interval. For example, if a participant’s monitoring window spanned 120 hours, but traffic was observed in only 100 of those hours, and their coverage rate was 83%. This definition captures transient lapses (eg, when the phone was rebooted or the VPN was manually disabled) while aligning with conventions in digital phenotyping, where adherence is measured as the percentage of expected intervals containing data [141-144].

At the day level, coverage was summarized using a complementary metric: a “valid day,” defined as any calendar day containing at least one active hour of VPN traffic. Whereas hour-level coverage quantifies the continuity of monitoring within a participant’s observed window, valid days capture the temporal span over which participants contributed data. Coverage was further examined across time-of-day and weekday and weekend strata to assess systematic gaps (see Multimedia Appendix 9 for stratification definitions).

#### Exploratory Analysis: Network Traffic

##### Inferring Behavioral Rhythms

To extract behavioral structure from encrypted network flows (ie, sequences of packets sharing a common source, destination, and time window), we treated traffic volume as a proxy for smartphone activity. Internet communication involves both downloads, which largely reflect content delivery (eg, high-volume video streams vs smaller text loads), and uploads, which more directly capture user-initiated actions such as taps, clicks, or navigation. Although exceptions exist—such as high upload traffic during calls or background updates—the relative balance between uploads and downloads provides a useful signal of interaction intensity. As phones are never completely silent, with low-level background exchanges for notifications and maintenance, we interpret higher-volume deviations above this baseline as more indicative of active user engagement.

To assess the validity of this assumption, we conducted an empirical evaluation using annotated sleep-wake periods on researcher devices, which showed systematically lower upload activity during sleep than wake periods (Multimedia Appendix 9).

In particular, we focus on percentile-normalized upload traffic as a heuristic proxy for user-initiated interaction because many common actions (eg, sending messages, posting, or searching) generate outbound requests. Raw byte counts were aggregated into contiguous, nonoverlapping 10-minute bins, a resolution that suppresses transient bursts while preserving diurnal dynamics. For each participant, this yielded a 24-hour activity vector per day. To account for differences in overall smartphone usage—arising from individual habits, app patterns, and device capabilities—we applied a rank-based, within-day percentile normalization. Specifically, for each day *d*, the traffic volume in bin *t* was replaced by its empirical percentile relative to the distribution {xd,1,...,xd,*T*}, where *T* denotes the number of bins in that day. This transformation is invariant to monotonic scaling and preserves intraday temporal structure, enabling direct cross-participant comparison of diurnal phase and amplitude while suppressing between-subject variance in absolute throughput.

**Figure 5.**
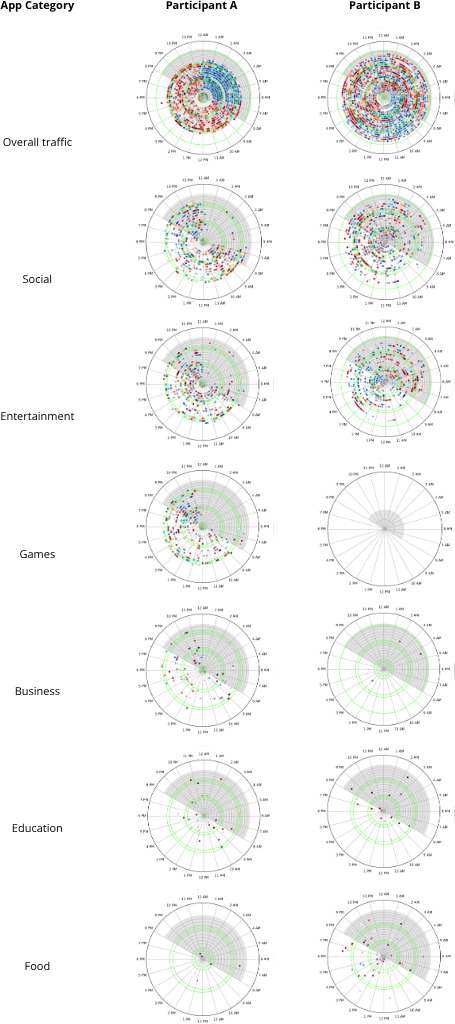
Temporal dynamics of overall and category-specific upload traffic for 2 representative participants. Each radial heatmap shows 24 h (angular axis) across successive days (concentric rings). Color indicates normalized traffic intensity, with red denoting higher device usage and blue denoting lower usage. Nighttime hours (8 PM to 8 AM) and weekends (green rings) are marked to facilitate alignment with social schedules.

We then visualized and analyzed normalized time series in 2 complementary ways. First, we projected daily activity onto a radial heatmap (Figure 5), where angular position denotes clock time (0‐24 h), concentric rings denote successive days, and color denotes normalized traffic intensity (red=high percentile and blue=low percentile). This polar representation highlights rest-activity cycles, phase shifts, and fragmentation of daily usage patterns. Nighttime intervals (8 PM to 8 AM) were shaded, and weekends were highlighted in green to facilitate alignment with social schedules.

To isolate context-specific rhythms, each network flow was enriched by mapping hostnames to high-level app categories (eg, social, entertainment, games, business, education, and food and drink). Mappings were derived from Apple’s APR (see section *Inferring Apps From Hostnames*), supplemented by manual verification and aggregation to category-level taxonomies. For each participant, we generated a set of paired radial visualizations: one depicting aggregate device activity and additional plots for each activity category of interest (Figure 5). This design enabled structured comparison between global digital behavioral rhythms and the distinct temporal patterns associated with multiple behavioral categories.

The result is a device-agnostic framework that combines upload-sensitive traffic features, percentile normalization, radial time encoding, and app-level categorization to detect time-of-day and behavioral regularities in digital behavior while preserving privacy. While some background traffic may still be misclassified as user activity and the precise boundary between passive and active phone use remains unresolved, we could still infer broad patterns of usage. More specifically, the approach identified meaningful diurnal and behavioral regularities inferred from encrypted network traffic alone, offering a scalable path for digital phenotyping without OS-level instrumentation.

##### Inferring Apps From Hostnames

###### Overview

Encrypted network traffic captured by our VPN consists of destination hostnames associated with network flows. While these hostnames provide a high-level view of device communication, they do not directly identify the apps responsible for generating traffic. In some cases, attribution is straightforward—for example, traffic to youtube.com almost certainly reflects YouTube usage. In many others, however, hostnames correspond to shared infrastructure such as content delivery networks, analytics services, or platform-level end points (eg, cloudflare.com and akamai.net), which are used by many unrelated apps. As a result, naïvely attributing hostnames to apps risks substantial misclassification.

Accurate app inference from network traffic, therefore, requires an external source of app-domain ground truth and a conservative strategy for handling ambiguity. In this study, our goal is not to exhaustively attribute all traffic to specific apps but rather to recover a reliable lower bound on app presence and timing that can support descriptive and exploratory behavioral analyses. To this end, we combine participant-collected and scripted app-domain observations and conservatively consider only domains that uniquely identify a single app.

The following subsections describe the data sources, filtering steps, and validation procedures used to construct this conservative app-domain mapping.

###### Data Sources: APR

To identify apps from observed hostnames, we leveraged Apple’s APR, an opt-in iOS feature that logs, at the app level, the set of hostnames contacted by each app over a rolling 7-day window. The APR is stored locally on the phone as a JSON file and provides a direct mapping between hostnames and the apps responsible for generating them.

APR data were collected from two complementary sources:

1. Participant-collected APRs. A subset of study participants elected to share their APR data. In total, APRs were collected from 15 participant devices, yielding app-domain observations spanning 247 distinct apps. These APRs reflect domains observed during naturalistic, real-world app use (“deep-state” usage).
2. Scripted APRs from controlled app interactions. To expand coverage beyond participant-reported apps and ensure representation of commonly used apps, we additionally collected APRs from controlled scripted interactions on research devices operated by the study team. On these devices, the study team manually interacted with 313 apps for approximately 1 minute per app, restricted to publicly accessible features and without creating user accounts for apps requiring authentication. Of these, 288 apps generated usable APR data and were included in the scripted APR corpus. App selection was informed by two sources: (1) 55 apps reported by participants in exit surveys and interviews and (2) top-ranked apps from the Apple App Store across multiple categories.

Together, participant-collected APRs (247 apps) and scripted APRs (288 apps) yielded a global union corpus spanning 424 distinct apps, with 111 apps observed in both datasets and comprising a total of 3788 registered domains. This unfiltered union corpus served as the starting point for the domain normalization and app-domain disambiguation procedures described below. To support higher-level behavioral analysis, apps identified via APR were mapped to functional categories using Apple’s iTunes Search API [145], which returns each app’s primary genre (eg, social, entertainment, and productivity). This yields a hierarchical mapping from hostname → app → behavioral category, illustrated in Figure 3.

###### Domain Normalization and Filtering

Raw hostnames recorded in APRs include a mixture of externally routable domains, local network identifiers, and nonroutable destinations such as IP literals. As only externally routable domains can be meaningfully associated with app backends or third-party services, we first filtered out local and nonroutable hostnames and then normalized the remaining hostnames to their externally routable registered (base) domains [146]. Full procedural details of domain filtering, normalization rules, and resulting domain counts are provided in Appendix I.1 in Multimedia Appendix 10.

This normalization step operates exclusively at the domain-entry level: individual hostname observations may be removed, but apps are not excluded at this stage. App-level exclusion occurs only during subsequent global app-domain disambiguation, where domains shared across multiple apps are conservatively removed to ensure unambiguous attribution (described below).

###### Global App-Domain Disambiguation

After domain normalization, many registered domains remained associated with multiple apps, typically reflecting shared infrastructure, such as content delivery networks, analytics services, advertising platforms, or OS-level services. As these domains cannot be reliably attributed to a single app, their inclusion would introduce ambiguity into app-level inference.

To ensure unambiguous attribution, we applied a conservative global app-domain disambiguation strategy over the union of participant-collected and scripted APR data. Only domains that uniquely mapped to a single app across the entire union corpus were retained; domains shared across multiple apps were excluded. For example, requests to youtube.com were retained because they appeared exclusively in association with the YouTube app, whereas domains such as cdn.apple.com or app-measurement.com, which appeared across many unrelated apps, were excluded. Under this global criterion, approximately two-thirds of registered domains (67.7%) were uniquely attributable to a single app, while the remaining domains (32.3%) reflected shared infrastructure and were conservatively excluded to avoid ambiguous attribution.

This conservative disambiguation substantially limits byte-level attribution, as shared infrastructure dominates overall traffic volume. Consistent with this expectation, only 1.47% of total upload bytes were attributable to app-unique domains. However, when evaluated temporally, 98.7% of 10-minute bins contained at least one app-attributable domain, indicating that app presence and activity timing are preserved across nearly all observation windows.

We therefore interpret app-level traffic attribution as a conservative lower bound on app usage volume. By prioritizing precision over coverage, this union-level procedure yields a reliable characterization of app presence suitable for feasibility assessment and exploratory behavioral rhythm analysis without relying on ambiguous infrastructure domains. Full disambiguation procedures and coverage statistics are provided in Appendix I.2 in Multimedia Appendix 10.

###### Scripted Versus Participant (“Deep-State”) Comparison

Brief scripted app interactions may not capture all domains that arise during prolonged or authenticated (“deep-state”) app use, raising concerns that scripted APRs could underrepresent app-identifying domains. To assess the impact of this limitation on app identification in our study, we compared domains observed during brief scripted launches with those observed during participant-collected, real-world usage of the same apps.

Despite differences in interaction depth, brief scripted launches recovered app-identifying domains for nearly the same set of apps as prolonged naturalistic use. Within the shared cohort, 30 of 47 apps were semantically identifiable based on domains observed during scripted interactions. Participant-collected (“deep-state”) usage identified only one additional app (31 in total), as domains observed during sustained or authenticated use offered limited incremental benefit and more often reflected shared infrastructure or generic services rather than app-specific signals.

These results indicate that, for the feasibility-oriented aims of this study, brief scripted interactions are sufficient to recover the app-identifying domains needed to infer app presence and activity timing. Full validation details—including app overlap criteria, semantic identifiability rules, and quantitative overlap statistics—are provided in Appendix I.3 in Multimedia Appendix 10.

### Ethical Considerations

This study was approved by the NYU Institutional Review Board (IRB-FY2025-9589) and conducted in accordance with the Belmont Report and the Declaration of Helsinki. All participants were adults and provided electronic informed consent prior to enrollment. No personally identifiable information or content data were collected; participants were assigned random identifiers, and traffic metadata were stored on secure, access-restricted university servers. Participants were compensated under an institutional review board–approved tiered schedule based on VPN connection duration, with payments issued via anonymized completion codes. No vulnerable populations were enrolled.

## Results

### Outcome 1: Feasibility

Feasibility was assessed using a mixed methods approach that combined quantitative measures of retention and data continuity with qualitative analysis of exit interviews. In this study, feasibility refers to participants’ ability to sustain passive VPN-based monitoring over time, as well as the operational conditions under which data collection was interrupted or discontinued.

#### Quantitative Feasibility: Retention and Data Coverage

##### User Retention

User retention was quantified as progression along the study pipeline (Figure 1). Specifically, we tracked the proportion of students advancing from initial eligibility screening through consent, onboarding, VPN activation, and study completion. This mirrors conventions in digital health feasibility studies, where recruitment yield and retention are reported alongside data completeness [147,148].

Recruitment outreach reached 100 students, of whom 38 provided informed consent. Of these, 32 completed the onboarding survey, 29 provided valid network traffic data, and 27 remained active for more than 5 days. At study completion, 26 participants completed the exit survey, and 25 completed the exit interview.

Overall, this corresponds to 93% retention among participants who contributed any valid network traffic data to the analysis stage (27/29; Wilson 95% CI 78%‐98%) and 66% completion from consent through the exit interview (25/38; Wilson 95% CI 50%‐79%). Comparisons between students who consented but did not continue and those who completed the study revealed no systematic differences in department, major, or university role, suggesting minimal self-selection bias that might confound usage patterns.

##### Data Coverage

Feasibility was also evaluated using data coverage, defined as the proportion of expected monitoring intervals with valid VPN traffic. As onboarding was staggered and the study ended on a fixed date, not all participants contributed a full 14 days of data. Feasibility was therefore defined relative to each individual’s observed monitoring window (from first to last active day), consistent with digital phenotyping conventions that measure adherence based on monitoring opportunity [149,150].

To ensure comparability of temporal rhythms, we restricted coverage analyses to the 24 participants who remained in a consistent time zone (ie, not traveling during the study period). Across these participants, the mean feasibility was 74.1% (median 77.1%; bootstrap 95% CI 66.3%‐81.4%), with coverage ranging from 21% to 100%. This variability reflects heterogeneous adherence, with some participants maintaining near-continuous VPN connectivity and others exhibiting intermittent gaps. On average, participants contributed 311.6 (~13 d, SD 3.5) hours of monitored traffic, with individual totals spanning 121 to 496 hours. In addition to overall coverage, we examined the temporal distribution of VPN off periods. Across all 24 participants, the median gap in coverage was 7.5 (IQR 2.8‐23.8) hours, with most interruptions being relatively short (ie, <24 h). However, occasional extended lapses occurred, with the maximum gap reaching 304 hours (~12.5 d). This extreme case (participant P05) showed nearly 13 days of continuous activity, followed by approximately 12.5 days of inactivity, before briefly reenabling the VPN at the study’s end.

Stratification confirmed that coverage was consistent across daily rhythms and calendar cycles (Table 1). The mean coverage was stable across mornings (71.1%), afternoons (75.9%), evenings (77.6%), and nights (71.9%), as well as between weekdays (74.2%) and weekends (74.0%). Aggregate analysis pooling bins across all participants yielded similar estimates (69.7%‐76.2% across strata; ), underscoring that passive VPN sensing did not systematically fail during specific daily routines or social contexts. On average, participants contributed 16.2 (SD 3.8, range 10‐23) valid days—defined as calendar days with at least one active hour of VPN traffic—reflecting the calendar span of participation across staggered onboarding and study completion.

Complementary visualizations illustrate continuity and adherence patterns. The calendar-aligned timeline (Figure G.1 in Multimedia Appendix 9) highlights deployment feasibility under staggered onboarding, while the Day-0–aligned timeline (Figure G.2 in Multimedia Appendix 9) normalizes trajectories to participants’ start dates, revealing heterogeneity in adherence and lapse patterns.

**Table 1.** Participant-level data coverage (mean and median across individuals), reported overall and stratified by time of day and weekday and weekend cycles.

Scope	Mean (%)	Median (IQR), (%)	95% CI (%)
Overall	74.1	77.1 (63.6-90)	62.7‐78.8
Morning	71.1	75.0 (57.9-88.6)	67.4‐83.3
Afternoon	75.9	79.0 (69.1-90.3)	70.1‐84.7
Evening	77.6	82.8 (67.1-92.4)	63.4‐79.7
Night	71.9	75.5 (56.8-85.8)	66.7‐81.7
Weekday	74.2	79.5 (63.5-86.6)	64.8‐83.2
Weekend	74	76.4 (61.2-99.4)	66.3‐81.4

### Qualitative Feasibility Findings

Participants’ accounts from the exit interviews provide a detailed picture of how the VPN-based monitoring system functioned in everyday use and why interruptions occurred. Across interviews, feasibility was most often described in practical terms—how easy the system was to set up, how much attention it required over time, and what happened when routine phone use or technical events disrupted monitoring. These findings reflect patterns that emerged across multiple rounds of independent coding and iterative synthesis, with themes developed to capture both facilitating and constraining conditions for feasibility.

Many participants began by describing onboarding as simpler than they had anticipated. Several entered the study expecting a technically demanding setup, only to find the process straightforward and clearly guided. As one participant reflected, “I thought this was going to be a lot more work, but it was really easy to participate in. I had no problems at all” (participant 24). Others emphasized the clarity of the instructions and the minimal learning required: “It was very clearly organized, so that made it effortless to set up” (participant 6) and “It’s just tapping one button—that’s it” (participant 10). For these participants, ease of setup shaped early expectations that participation would be manageable alongside academic and personal routines.

Once installed, participants consistently described the system as requiring little to no ongoing effort. Rather than needing active maintenance or frequent checking, the VPN was described as something that simply “ran” while participants went about their normal activities. One participant explained, “There was nothing too complex. It wasn’t something I had to maintain or keep monitoring” (participant 7), while another noted, “It was very little effort—pretty invisible” (participant 22). This low operational demand appeared central to sustained participation. Several participants linked their continued enrollment to the fact that the system did not demand much ongoing effort: “At first I thought it would be more hands-on, but in the end I didn’t really have to do much” (participant 18).

For some participants, the system’s unobtrusiveness was so pronounced that it faded entirely from awareness. Multiple interviewees described forgetting about the VPN altogether after initial setup. As one participant put it, “I set it up and then just forgot about it, basically” (participant P3). Another participant echoed this sentiment: “It was one of those things where you forget about it” (participant 12). In several cases, this invisibility led participants to continue running the VPN beyond the planned 14-day study period simply because there was no reason to turn it off. These accounts align with objective data showing that some participants contributed more than 2 weeks of valid recordings without deliberate effort to sustain participation.

Participants also emphasized that the system did not interfere with daily life. Many described being able to use their phones normally, without changes to work, study, or leisure activities. One participant stated plainly, “It didn’t interfere at all with anything” (participant P6), while another explained, “I could still use my phone the way I was used to—I didn’t really notice it throughout the day” (participant 14). For these participants, feasibility was closely tied to the system’s ability to blend into existing routines rather than impose new ones.

At the same time, this same invisibility contributed to several of the feasibility breakdowns observed in the quantitative data. As the VPN demanded so little attention, participants often failed to notice when monitoring stopped. The most common scenario involved phones powering off, batteries dying, or devices restarting. Several participants described realizing only later that data had not been collected during these periods. One recalled, “My phone died and I didn’t realize the VPN turned off. It took almost a day before I noticed” (participant 6). Another participant explained, “I didn’t know that once my phone died I would have to manually turn it back on” (participant 7).

Importantly, participants were clear that these interruptions were not intentional. When asked directly, several stated that they had never deliberately disabled the VPN: “No, I never turned it off intentionally at all” (participant 24). Instead, lapses were described as the result of forgetting that the VPN required manual reactivation after device-level events. In this sense, the same low-maintenance design that supported feasibility also reduced participants’ awareness of the system state.

A smaller number of participants described intentional pauses in monitoring due to perceived technical side effects. These included concerns about battery drain during long days away from charging or frustration with occasional network slowdowns. One participant explained, “When videos took a long time to load, I’d get frustrated and turn off the VPN for a bit” (participant 10), while another participant noted, “I noticed my battery draining really fast” (participant 7). Although these cases were relatively uncommon, they tended to correspond to longer gaps in data coverage. Importantly, independent laboratory measurements indicated that the VPN consumed less than 1% battery over a 24-hour period, suggesting that these interruptions were driven less by objective resource usage than by participants’ perceptions of device performance.

Finally, interviews revealed that participants’ perceptions of their own adherence did not always align with recorded data. Some participants overestimated how long the VPN had been active, while others underestimated it. As one participant reflected uncertainly, “I want to say it was like two and a half weeks...” (participant 2). These discrepancies highlight the limits of self-report for assessing feasibility and reinforce the value of passive logging in understanding real-world use.

### Outcome 2: Acceptability

Acceptability was evaluated using a mixed methods approach combining standardized workload and usability instruments with in-depth qualitative analysis of exit interviews. In this study, *acceptability* refers not only to whether participants were willing to tolerate the system but also to their perceptions of its appropriateness, trustworthiness, and value for sustained use in everyday life.

#### Quantitative Acceptability: Workload and Usability

To evaluate the acceptability of our system in naturalistic daily use, we administered adapted versions of the NASA-TLX [117] and the SUS [132] during the exit survey. These instruments quantify, respectively, the perceived workload associated with system use and the overall usability of the interface and interaction design.

As shown in Figure G.3 in Multimedia Appendix 9, NASA-TLX ratings indicated consistently low levels of burden across all 6 dimensions: mental demand (mean 2.69, SD 3.27), physical demand (mean 1.54, SD 1.79), temporal demand (mean 2.92, SD 4.09), performance (mean 4.08, SD 4.18), effort (mean 3.85, SD 4.31), and frustration (mean 2.88, SD 3.78). The boxplots display the distribution of responses, with medians (horizontal lines) often below the mean values (black dots), reflecting skewness due to a small number of outliers (open circles). Although the NASA-TLX does not prescribe thresholds for “acceptable” workload, the uniformly low scores suggest that participants experienced minimal cognitive, physical, or temporal strain.

For context, prior digital health deployments have reported substantially higher NASA-TLX scores—for example, a COVID-19 exposure notification app showed a mean mental demand of approximately 29 out of 100 and overall workload of 25 to 35 out of 100 [106]. In contrast, our participants’ scores corresponded to approximately 10 to 20 out of 100, indicating that the VPN-based system operated unobtrusively with minimal daily burden.

Additionally, SUS results reinforced this conclusion. The mean SUS score was 78.08 (SD 14.96), ranging from 52.5 to 100. According to established SUS benchmarks [151], scores above 68 are interpreted as reflecting good usability, positioning our passive monitoring system well above the benchmark for acceptability.

#### Qualitative Acceptability in Everyday Use

Participants’ interview accounts provide deeper insight into how these low workload and high usability scores translated into lived experience. Across interviews, acceptability was often articulated in practical terms, including the degree of perceived intrusiveness, alignment with participants’ expectations around privacy and control, and the perceived value of the system beyond data collection.

Many participants framed their acceptance of the system around its minimal impact on daily life. The VPN was frequently described as something that “just ran” without requiring attention or disrupting routines. As one participant noted, “It was very little effort. Pretty invisible” (participant 22). Another participant explained, “It didn’t interfere at all with anything” (participant 6). For these participants, acceptability was closely tied to the absence of friction: the system did not ask them to change how they used their phones or manage new tasks.

Trust and institutional context also played a central role in acceptability. Several participants described feeling comfortable with passive monitoring because they trusted the research team and the university affiliation. Clear explanations during onboarding about what data were collected, how they were handled, and how anonymity was preserved helped establish this trust. As 1 participant reflected, “You explained it pretty well—what you were doing, how you were capturing the data, how you were making sure it wasn’t attached to my name” (participant 14). This transparency appeared to reduce anxiety and supported continued participation even when monitoring was largely invisible.

At the same time, acceptability was not unconditional. A subset of participants expressed ongoing discomfort during specific activities, particularly those involving financial or sensitive personal information. Three participants described feeling uneasy during banking or similar tasks, and 7 participants reported occasionally disabling the VPN in such contexts. One participant explained simply, “Sometimes I turn it off when I’m using my banking apps” (participant 20). These behaviors illustrate that acceptability was negotiated dynamically: participants balanced trust in the system with situational judgments about privacy and control.

Importantly, these selective interruptions did not reflect wholesale rejection of the system. Rather, they demonstrate how participants exercised agency to manage perceived risk while remaining broadly willing to participate. For most participants, the ability to turn the VPN off when desired appeared to *increase* acceptability by preserving a sense of control.

Acceptability also extended beyond tolerance to perceived benefit for some participants. Several described the dashboard and visualizations as prompting reflection on their own digital behaviors. Twelve participants reported increased awareness of smartphone usage patterns, with a subset noting intentional behavior changes as a result. One participant observed, “Seeing the patterns in my phone usage on the heat map was interesting. It made me more aware of how much I use my phone, and sometimes I felt like I might need to tone it down” (participant 11). Another described realizing how quickly time passed during app use: “It made me notice just how quickly 40 minutes can disappear” (participant 2). In some cases, this awareness motivated short-term changes, such as reducing social media use: “Once I saw it was tracking my activity, I actually took a break from Instagram for a week” (participant 13).

These perceived benefits contributed to the willingness to continue. Nineteen participants expressed interest in using the system beyond the 14-day study period, describing it as unobtrusive and easy to live with. Four others expressed a neutral but permissive stance—“no reason to stop using it”—reflecting that the system imposed little cost even if it did not actively engage them. Others indicated that continued use would depend on resolving specific reliability concerns, particularly around unexpected VPN shutdowns.

### Outcome 3 (Exploratory): Capturing Behavioral Rhythms From Network-Derived Features

To explore patterns of daily structure in passive network traffic relevant to behavioral rhythms, we conducted an exploratory, cohort-level analysis using interpretable, participant-level traffic-derived rhythm metrics, as described below.

#### Cohort-Level Rhythm Metrics

For each participant, we derived 2 complementary metrics that summarize daily activity patterns from upload network traffic after within-day rank (percentile) normalization: (1) day-to-day stability, computed as the mean correlation between consecutive daily activity profiles, following prior work on cross-day behavioral regularity [152]; and (2) circadian power ratio, defined as the proportion of spectral power concentrated at the 24-hour frequency and serves as a standard spectral measure of circadian rhythmicity [153,154].

**Figure 6.**
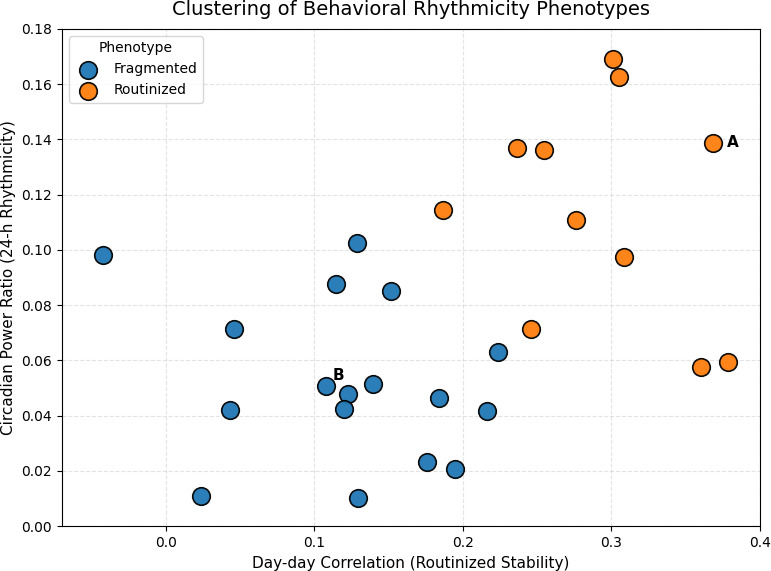
Cohort-level behavioral rhythm structure derived from passive network traffic. Each point represents 1 participant, plotted by day-to-day activity stability and circadian power ratio. Colors indicate unsupervised clustering performed for descriptive purposes. Participants A and B are annotated to illustrate how exemplar temporal profiles (Figure 5) relate to cohort-level structure.

Figure 6 shows the joint distribution of these metrics across all participants. Together, these metrics capture complementary aspects of behavioral regularity: consistency across days (day-to-day stability) and strength of circadian rhythmicity (circadian power ratio). To identify dominant behavioral patterns across participants without imposing predefined thresholds, we applied unsupervised clustering in the joint space of these 2 metrics. Specifically, we performed k-means (k=2) clustering on z score–standardized values of day-to-day stability and circadian power ratio to ensure comparable scaling across metrics. We selected 2 clusters as a minimal and interpretable partition aligned with the dominant structure observed in the joint metric space, separating participants with relatively more routine versus more fragmented activity rhythms. This analysis revealed 2 broad, data-driven groupings corresponding to relatively more routine versus more fragmented activity rhythms across participants.

#### Illustrative Participant-Level Temporal Patterns

Two participants (A and B) are annotated in Figure 6 as occupying distinct regions of the cohort-level rhythm space, reflecting contrasting patterns of behavioral regularity and fragmentation. To illustrate how these cohort-level differences manifest in individual behavior, Figure 5 presents detailed temporal visualizations for these participants.

Participant A exhibited a highly regular daily pattern, with activity concentrated during daytime hours and minimal activity overnight. The radial plots show a strong and stable day-night rhythm across days, with social media activity closely mirroring overall traffic. Category-specific views further indicate consistent engagement with gaming and business-related apps, alongside limited interaction with food delivery services.

In contrast, participant B displayed a more fragmented and irregular activity profile. Although some day-night structure was present, social media activity appeared at variable and atypical hours, including late night and early morning. Category-level traces showed comparatively greater reliance on food-related services and minimal engagement with gaming or business apps.

Figure 6 shows cohort-level variation in behavioral regularity alongside participant-level temporal patterns derived from encrypted network traffic. The 2 participants are presented as illustrative examples situated within the broader cohort-level structure, rather than as representative or diagnostic cases. This analysis is explicitly exploratory and descriptive and does not claim inferential separation or validated behavioral phenotypes; instead, it is intended to demonstrate the feasibility of deriving interpretable rhythm features from passive network data.

## Discussion

### Principal Results

This study examines encrypted smartphone network traffic as a means of longitudinally characterizing the temporal organization of everyday digital activity under real-world conditions. Rather than inferring specific behaviors or clinical states, we focus on how passive network metadata captures interpretable temporal patterns in digital activity, including periods of sustained activity, relative inactivity, and variability in daily engagement. Such temporal structure has been widely studied in health and behavioral research as a marker of routine organization and change but is difficult to observe continuously without intrusive or burdensome sensing approaches [155,156]. Accordingly, we interpret our findings in terms of their implications for measuring behavioral rhythms at scale, rather than for identifying specific health conditions or outcomes.

Empirically, we observe that encrypted network traffic exhibits interpretable temporal structure within individuals, characterized by recurring patterns of activity and inactivity over time. While the degree of regularity varied across participants, these temporal patterns could be recovered without inspecting content or requiring intrusive sensing. Using a VPN-based monitoring system, we sustained continuous data capture for nearly 2 weeks on average, with minimal participant effort, demonstrating that longitudinal observation of everyday digital activity is feasible in naturalistic settings without intrusive sensing or content inspection.

From the perspective of studying behavioral organization over time, sustained feasibility is critical, as changes in routine structure and engagement often unfold over weeks rather than days [1-3,157]. In descriptive comparison with prior remote digital health studies reporting median retention of approximately 5.5 days or completion rates near 50% [147,158], our 93% retention among contributing participants (27/29, 93.1%) lies at the upper end of reported values, suggesting that network-level monitoring can sustain multiweek observation windows sufficient to capture day-to-day variability and gradual change.

Beyond retention, participant accounts indicate that feasibility depended on a balance between minimal operational burden and low system salience—conditions that are essential for observing naturalistic patterns of everyday behavior. Participants consistently described the system as lightweight and unobtrusive, with SUS scores well above standard usability benchmarks, and interview accounts highlighting its “invisible” operation in daily life. This low level of disruption is particularly important for studies aimed at characterizing routine organization and temporal regularity, as more intrusive monitoring can itself alter behavior. Coverage gaps arose primarily from device-level interruptions, benign senescent forgetfulness [159], or occasional technical frictions rather than resistance to participation or perceived burden.

Acceptability was shaped not only by low operational burden but also by privacy, trust, and perceived control. Most participants expressed confidence in the research team and institutional safeguards; when concerns arose, they were typically addressed through selective, temporary disabling of the VPN during sensitive activities rather than disengagement from the study. Importantly, this pattern suggests that acceptability was not passive but actively negotiated over time, with user control enabling continued participation even in the presence of situational discomfort.

For behavioral and health-oriented research, this capacity for negotiation is consequential: rigid, always-on sensing can provoke disengagement, whereas approaches that allow situational control may better support long-term observation of everyday routines. Participants’ qualitative accounts aligned closely with quantitative indicators of low workload and high usability, reinforcing that acceptability was actively maintained throughout the deployment.

Exploratory analyses further indicated that traffic-derived features captured coherent daily patterns of digital activity rhythms, with systematic variation across participants in temporal regularity and routine structure. Specifically, individuals differed in the consistency, concentration, and fragmentation of activity over the course of the day, reflecting variation in how daily routines were organized. While this study does not assess specific behaviors or health outcomes, the ability to recover stable, individual-level differences in behavioral regularity demonstrates that encrypted network traffic can serve as a viable proxy for longitudinal study of everyday behavioral organization. Such dimensions of behavioral organization have been widely studied in health and behavioral research as early indicators of changes in mental and cognitive health, even before clinical diagnosis [100-104].

### Limitations and Future Work

Several limitations constrain interpretation and generalizability. Network traffic alone cannot reliably distinguish foreground user interaction from background processes, as updates, notifications, and keep-alive signals may generate activity without active use. Although percentile normalization reduces some noise, it remains a coarse proxy for engagement rather than a definitive separation of foreground and background states.

**Figure 7.**
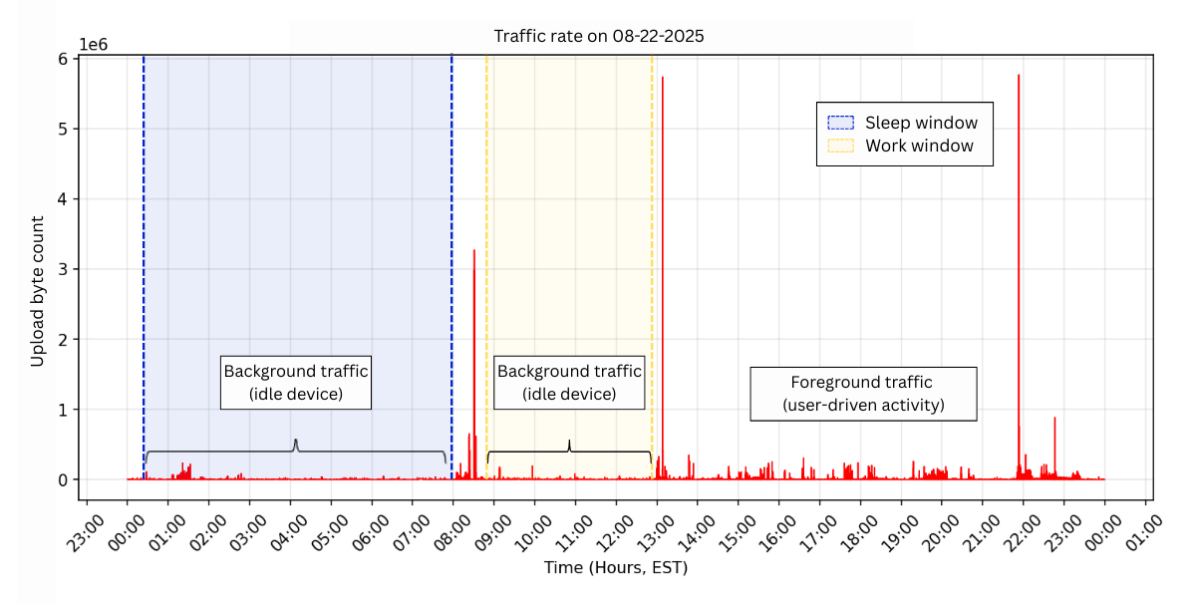
Daily upload traffic from a researcher’s device on August 22, 2025. Background periods (eg, sleep and work windows) are shaded, with bursts of foreground activity visible as spikes in upload volume.

As illustrated in Figure 7 and supported by the empirical evaluation in Multimedia Appendix 9, sleep periods exhibited lower percentile-normalized upload traffic than awake periods, with sharp peaks more likely reflecting user-driven activity. However, background processes can occasionally mimic bursty patterns, and these distinctions should therefore be interpreted as heuristic approximations. Additionally, not all behaviors generate network traces. Fully offline actions—such as composing notes without iCloud sync or using local-only apps—cannot be observed through this approach. While this limits the ability to attribute activity to specific behaviors, it also serves as a privacy safeguard by reducing the risk of overidentification while still capturing meaningful behavioral rhythms.

Another limitation concerns the temporal and platform stability of app-domain mappings derived from Apple’s APR, which exposes app-domain associations over a rolling 7-day window, whereas our monitoring period extended to approximately 2 weeks. Our analyses, therefore, assume that app-identifying domains remain sufficiently stable over short deployments. Although we did not explicitly measure temporal drift, the consistency of domains recovered across participant-collected APRs and controlled scripted interactions suggests that the retained mappings reflect persistent backend infrastructure rather than transient connections. Over longer deployments, however, app updates or changes in third-party services may necessitate periodic reestimation. Moreover, APR is currently limited to iOS; extending this approach to Android will require alternative strategies such as controlled app interaction experiments, longitudinal aggregation, or hybrid labeling pipelines.

From a systems perspective, data gaps following phone restarts occasionally led to extended lapses in monitoring. These gaps highlight the need for stronger reliability safeguards. Future iterations should prioritize automated VPN reactivation and user-facing notifications when connectivity lapses occur, ensuring continuous coverage without manual intervention.

Beyond technical considerations, awareness of being monitored may introduce reactivity effects. While several participants reported forgetting about the system entirely, others used the feedback productively to reflect on their digital habits. Future work should distinguish between distortion of natural behavior and constructive self-awareness, clarifying when monitoring alters behavior in ways that undermine inference versus when it provides meaningful benefit.

Generalizability is also limited by the demographic composition of our sample, which consisted of university students from a single elite US institution—a group likely to be more familiar with VPNs and digital privacy trade-offs. Although technology adoption among older adults is increasing [160-167], comfort with passive monitoring and mental models of digital surveillance may differ substantially across populations. Broader recruitment will be essential for evaluating feasibility and acceptability in more diverse and representative cohorts.

Looking ahead, an important direction for future research is to evaluate the utility of network-derived behavioral rhythms in populations at elevated risk for cognitive and neuropsychiatric disorders. Prior work has shown that disruptions in sleep-wake cycles and daily behavioral regularity often precede clinical diagnosis of conditions such as depression, Alzheimer disease, and Parkinson disease. While this study was not designed to assess clinical outcomes, its contribution lies in establishing a low-burden, scalable method for measuring the longitudinal behavioral patterns that such studies aim to track in aging or clinical populations.

Equally important is validation against independent ground truth. Combining network traffic–derived rhythms with complementary data sources—such as accelerometer signals, wearable-derived sleep measures, location context, or experience sampling—would enable stronger links between digital activity patterns and behavioral or psychological states. Cross-device monitoring represents another promising extension. As WireGuard is compatible with all major operating systems, expanding monitoring to laptops and tablets could provide a more holistic view of how individuals transition between contexts throughout the day.

Finally, methodological refinements will be necessary to capture finer-grained app usage. Preliminary experiments suggest that different functionalities (eg, text messaging vs video calls) produce distinct network signatures. Systematic labeling in controlled settings, paired with user-reported logs, could enable classifiers to infer more specific behaviors at scale. Together, these extensions—broader populations, cross-device integration, multimodal validation, and analytic refinement—would move the system beyond feasibility and acceptability toward a robust, generalizable platform for longitudinal behavioral monitoring, with apps ranging from circadian rhythm research to cognitive health.

### Relation to Prior Work

Our method occupies a distinct space between high-burden active sensing and coarse, platform-restricted analytics. Existing approaches—including self-reports, OS-level logs, and custom sensing apps—face challenges such as recall bias, declining adherence, battery overhead, and restrictive platform permissions, limiting their ability to support longitudinal observation in naturalistic settings.

We address these limitations in 3 ways. First, the system is OS-agnostic: operating at the network layer via a standard VPN profile, it functions across iOS, Android, laptops, and other devices without rooting or platform-specific APIs. Second, it preserves privacy by capturing only metadata (eg, timestamps and encrypted flows) rather than message content or typed input. Third, it incorporates participant-facing dashboards that enhance transparency and support informed participation.

Together, this combination of precision, privacy preservation, and scalability differentiates our method from prior sensing paradigms and establishes it as a flexible foundation for behavioral research in naturalistic, longitudinal settings.

### Conclusions

Our study demonstrates the feasibility and acceptability of a passive monitoring system for encrypted network traffic that can be mapped into interpretable activity patterns. By sustaining unobtrusive capture over extended periods, the approach yields rhythms aligned with circadian structure and digital routines while minimizing participant burden. Limitations in app-level inference, demographic scope, and device coverage define clear avenues for refinement. With broader recruitment, multidevice integration, and validation against ground truth, this system could expand the digital phenotyping toolkit for investigating sleep, mental health, and cognitive change across the lifespan at scale.

## Supplementary material

10.2196/84618 Multimedia Appendix 1 Demographic characteristics of the final analytic sample.

10.2196/84618 Multimedia Appendix 2 Recruitment and onboarding procedures.

10.2196/84618 Multimedia Appendix 3 VPN implementation and data collection details.

10.2196/84618 Multimedia Appendix 4 Participant onboarding and study portal.

10.2196/84618 Multimedia Appendix 5 Data capture and participant portal specifications.

10.2196/84618 Multimedia Appendix 6 Semistructured interview questions.

10.2196/84618 Multimedia Appendix 7 Exit survey administration and interview procedures.

10.2196/84618 Multimedia Appendix 8 Qualitative codebook summarizing themes, subthemes, and original codes derived from exit interview transcripts.

10.2196/84618 Multimedia Appendix 9 Coverage stratification and aggregate feasibility.

10.2196/84618 Multimedia Appendix 10 App-domain inference pipeline and validation.

## Abbreviations

AI: artificial intelligence
API: application programming interface
APR: App Privacy Report
CONSORT: Consolidated Standards of Reporting Trials
GAIDeT: Generative Artificial Intelligence Delegation Taxonomy
NYU: New York University
OS: operating system
SUS: System Usability Scale
VPN: virtual private network
