# Supplementary material for: Digital Phenotyping via Passive Network Traffic Monitoring: Prospective Observational Study in University Students
Source: JMIR Form Res. 2026 Apr 27;10:e84618. doi: 10.2196/84618 (PMC13118141; doi:10.2196/84618)
Supplement: Multimedia Appendix 1 [file formative-v10-e84618-s001.docx]

### Demographic Characteristics of the Final Analytic Sample

Demographic characteristics of the final sample are summarized in Table J.1. Exit interviews were conducted with 25 participants, who reflected on their experiences using our system, reported perceived benefits and burdens, and provided usability feedback. The sample comprised 12 women, 12 men, and one non-binary participant. The mean age was 22 years (SD = 2.9; range 18–29). Educational levels spanned undergraduates (n = 20), one master’s student, and four doctoral students, representing a variety of majors. The most common fields were Chemistry (6), Economics (5), Computer Engineering/Science (5), Data Science (3), and Mathematics (3). Racial and ethnic composition included Asian (13), White/European (6), Latin/Hispanic (3), Black/African/Caribbean (3).

| **Demographic Variable** | **Category/Description** | **Count** | **%** |
| --- | --- | --- | --- |
| Age | Mean (SD) | 22.04 (2.95) |  |
|  | Range | 18-29 |  |
| Gender | Male | 12 | 48.0% |
|  | Female | 12 | 48.0% |
|  | Non-binary | 1 | 4.0% |
| Race/Ethnicity | Asian | 13 | 52.0% |
|  | White/European | 6 | 24.0% |
|  | Latin/Hispanic | 3 | 12.0% |
|  | Black/African/Caribbean | 3 | 12.0% |
| Degree Level | Undergraduate | 20 | 80.0% |
|  | Master’s | 1 | 4.0% |
|  | Ph.D. | 4 | 16.0% |
| Major | Chemistry | 6 | 24.0% |
|  | Computer Engineering | 3 | 12.0% |
|  | Economics | 3 | 12.0% |
|  | Biochemistry | 2 | 8.0% |
|  | Data Science | 2 | 8.0% |
|  | Others (9 majors) | 9 | 36.0% |

Table J.1. Demographic characteristics of interview participants (N = 25), summarizing age, gender, race/ethnicity, degree level, and field of study.
