# Supplementary material for: Digital Phenotyping via Passive Network Traffic Monitoring: Prospective Observational Study in University Students
Source: JMIR Form Res. 2026 Apr 27;10:e84618. doi: 10.2196/84618 (PMC13118141; doi:10.2196/84618)
Supplement: Multimedia Appendix 2 [file formative-v10-e84618-s002.docx]

### Recruitment and Onboarding Procedures

#### A.1 Recruitment Rationale

Participants were recruited through academic advisors across multiple New York University schools and departments. This advisor-mediated approach was used because the university does not maintain a centralized student mailing list. This decentralized strategy leveraged existing academic networks to reach a diverse student population relevant to our focus on young adult digital behaviors. It also enabled outreach to students across a range of academic disciplines, including STEM, arts, and social sciences. Recruitment materials described the study at a high level (i.e. screen-time behaviors) and directed interested students to an opt-in sign-up form; researchers did not directly contact students prior to opt-in.

#### A.2 Recruitment Timeline

Initial outreach was conducted in April 2025 via advisor-distributed invitations. Interested students received a consent packet in mid-May 2025, after which enrollment and onboarding occurred on a rolling basis. Participants who completed onboarding participated in a two-week passive monitoring period.

#### A.3 Consent and Enrollment Workflow

Interested students received a consent packet describing the study purpose, passive monitoring procedures, potential privacy risks, and participant rights, including the ability to withdraw at any time without penalty. Consent was obtained electronically via a secure web portal. Upon providing consent, participants were immediately directed to the onboarding workflow.

#### A.4 Onboarding and Technical Setup

Onboarding was conducted through a secure web portal that guided participants through installation and configuration of the WireGuard virtual private network (VPN) application. Participants were instructed to activate the VPN during the study period to enable passive traffic collection.

#### A.5 Inclusion and Exclusion Considerations

Eligibility criteria included being 18 years of age or older and able to provide informed consent independently, the ability to complete study surveys and interviews in English, and residence in a household with a dedicated, independent home Wi-Fi router (i.e., not a shared building-level network). Participants were also required to have access to a compatible personal device capable of running the WireGuard application.

Participants were excluded if they did not meet the above inclusion criteria or if technical or screening constraints prevented successful onboarding.
