# Supplementary material for: Digital Phenotyping via Passive Network Traffic Monitoring: Prospective Observational Study in University Students
Source: JMIR Form Res. 2026 Apr 27;10:e84618. doi: 10.2196/84618 (PMC13118141; doi:10.2196/84618)
Supplement: Multimedia Appendix 3 [file formative-v10-e84618-s003.docx]

### VPN Implementation and Data Collection Details

We implemented VPN-based passive traffic monitoring using the WireGuard protocol [116], which provides a lightweight mechanism for capturing device-to-internet traffic metadata without modifying the mobile operating system or requesting intrusive permissions. By design, a VPN forwards all device-to-internet communication through an intermediary server. Without a VPN, a device exchanges encrypted packets (i.e., small units of transmitted data) directly with destination servers. When the VPN is active, these encrypted packets are first transmitted to the VPN server, which then relays them to the intended destinations and returns responses to the device. Although packet contents remain end-to-end encrypted, the VPN operator can observe traffic metadata in real time, including contacted hostnames (domain names of services), packet counts, and byte volumes.

Among available VPN protocols (e.g., L2TP, IPsec, OpenVPN), we selected WireGuard for its simplicity, cross-platform availability (iOS, Android, macOS, Windows), and robustness under continuous operation. To minimize participant burden, configuration errors, and perceived intrusiveness—and to foster participant trust—we used the official WireGuard client rather than developing a custom application. The WireGuard client is widely distributed through platform app stores and consistently ranks among the most reliable VPN solutions.

Participants installed the WireGuard client directly from the appropriate app store and configured it by scanning a personalized QR code generated by the study server hosted at NYU. This process required no manual network configuration and automatically routed all device-to-internet traffic through a study-managed VPN endpoint, making onboarding nearly frictionless.

On the server side, traffic was routed through a Linux-based VPN server, where metadata were captured using the open-source packet analysis tool tshark [117]. Packet contents were neither inspected nor stored. Instead, captured packets were aggregated into fixed 10-second windows. For each participant and time window, we retained the following metadata fields: an anonymized participant identifier (PID), timestamp, contacted hostname, number of packets sent and received, and byte volumes transmitted in each direction. These metadata served as input to downstream processing pipelines that mapped hostnames to mobile applications and activity categories, as described in the main Methods section.

Captured metadata were stored on NYU-managed infrastructure with restricted access. Figure 1 in the main text illustrates the overall system architecture, in which all device-to-internet traffic is routed through the WireGuard VPN server. While message contents remain end-to-end encrypted, the server captures traffic metadata (e.g., hostnames and traffic volumes), enabling passive behavioral analysis while preserving content privacy.

WireGuard demonstrated strong reliability in handling routine connectivity disruptions, including Wi-Fi–cellular handoffs and airplane mode toggles, and produced fewer data gaps than alternative VPN clients evaluated during pilot testing. Energy efficiency was also a key consideration. In 24-hour continuous-use tests conducted on a laboratory iPhone running iOS 18, WireGuard consumed less than 1% of total battery capacity. Battery impact was assessed by comparing charge depletion rates with and without the VPN enabled, indicating that participants could keep the VPN active indefinitely without noticeable effects on device usability.
