# Supplementary material for: Digital Phenotyping via Passive Network Traffic Monitoring: Prospective Observational Study in University Students
Source: JMIR Form Res. 2026 Apr 27;10:e84618. doi: 10.2196/84618 (PMC13118141; doi:10.2196/84618)
Supplement: Multimedia Appendix 4 [file formative-v10-e84618-s004.docx]

### Participant Onboarding and Study Portal

After enrollment, participants were directed to a secure online portal to complete study setup. During this process, participants generated a cryptographically random participant identifier (PID). If desired, participants could regenerate their PID until satisfied. Once finalized, participants were instructed to store the PID securely, as it was required for re-entry to the portal and could not be recovered if lost.

Participants were then instructed to download the official WireGuard VPN application from the iOS or Android app store. The portal generated a unique QR code embedding the study-specific VPN configuration. Participants scanned this QR code within the WireGuard app, which automatically configured their device to route all internet traffic through the NYU-managed study VPN server.

The portal dashboard confirmed successful installation and connection status before participants proceeded to the data collection phase. This verification step ensured that VPN routing was correctly established prior to the start of monitoring.

Appendix Figure C.1 shows the participant-facing onboarding portal and QR-based setup flow used during the study.

Each participant scanned a unique QR code to automatically configure the WireGuard VPN client and route traffic through the study server.


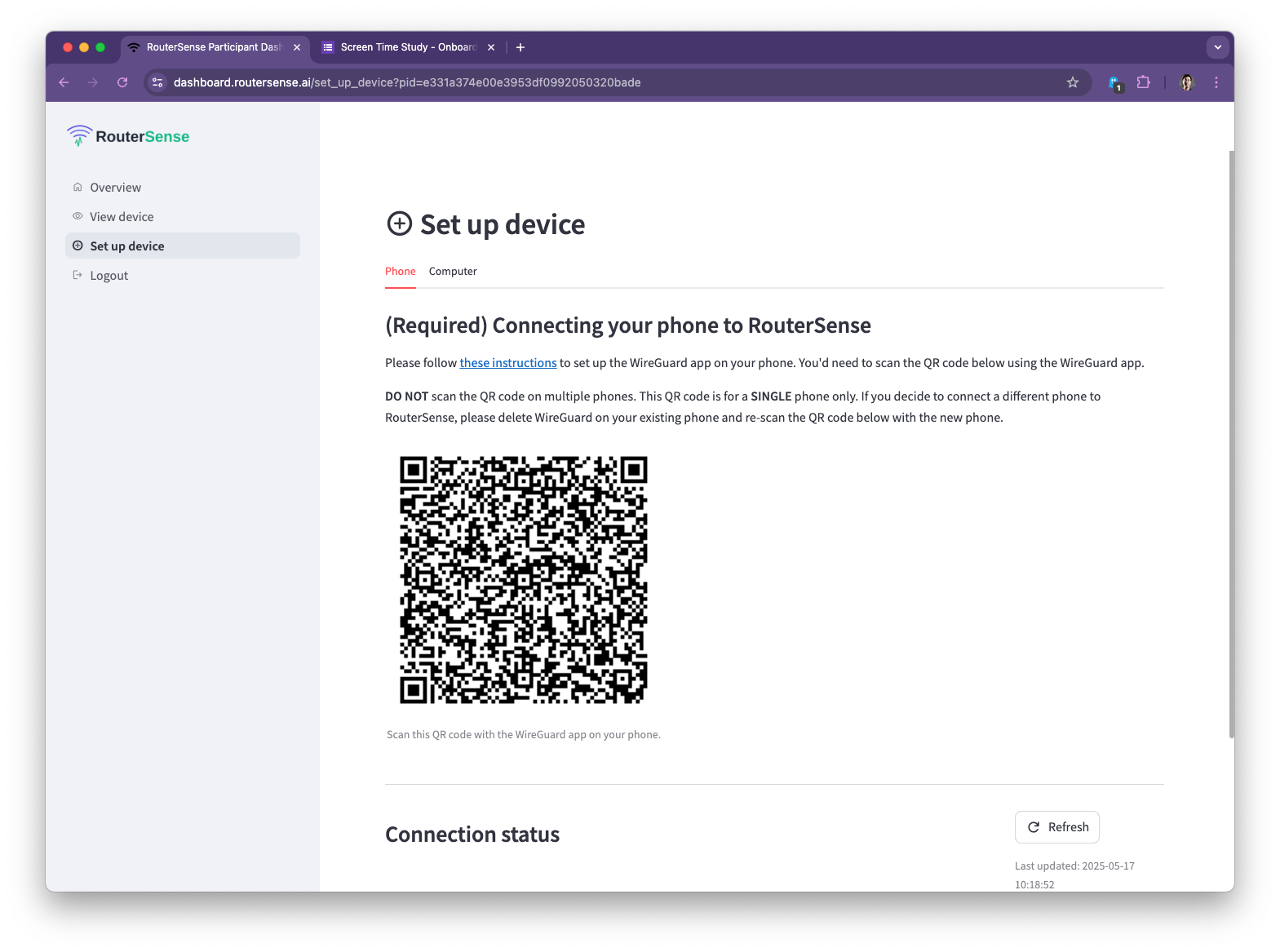


Figure C.1. Participant onboarding portal. Each participant scanned a unique QR code to automatically configure the WireGuard VPN client and route traffic through the study server.
