# Supplementary material for: Digital Phenotyping via Passive Network Traffic Monitoring: Prospective Observational Study in University Students
Source: JMIR Form Res. 2026 Apr 27;10:e84618. doi: 10.2196/84618 (PMC13118141; doi:10.2196/84618)
Supplement: Multimedia Appendix 5 [file formative-v10-e84618-s005.docx]

### Data Capture and Participant Portal Specifications

Traffic metadata were aggregated on the VPN server in fixed 10-second windows. For each window, the following fields were recorded: anonymized participant identifier (PID), timestamp, destination hostname, packet counts (uplink/downlink), and byte volumes (uplink/downlink). Packet payloads were never inspected or stored. Metadata were written to a secure SQLite database hosted on NYU-managed infrastructure with restricted access.

VPN inactivity could occur due to manual disablement or device reboot. Reminder emails were sent every 2–3 days prompting participants to verify VPN status via the study portal. The portal queried server-side logs to compute cumulative active study days and compensation.

Compensation followed a tiered structure. Participants received $0 for fewer than five cumulative days of VPN activity, $38 for 5–7 days, $39 for 7–10 days, $40 for 10–14 days, and $41 for more than 14 days. This structure was deliberately designed to encourage sustained participation beyond the initial five-day warm-up period while minimizing the influence of financial incentives on longer-term engagement. The incremental increases in payment were deliberately small to limit the influence of financial incentives on participation, since the marginal benefit of continued engagement diminished over time. Rolling recruitment meant that the overall study period—from the first participant onboarded to the last participant exited—spanned approximately 20 days.


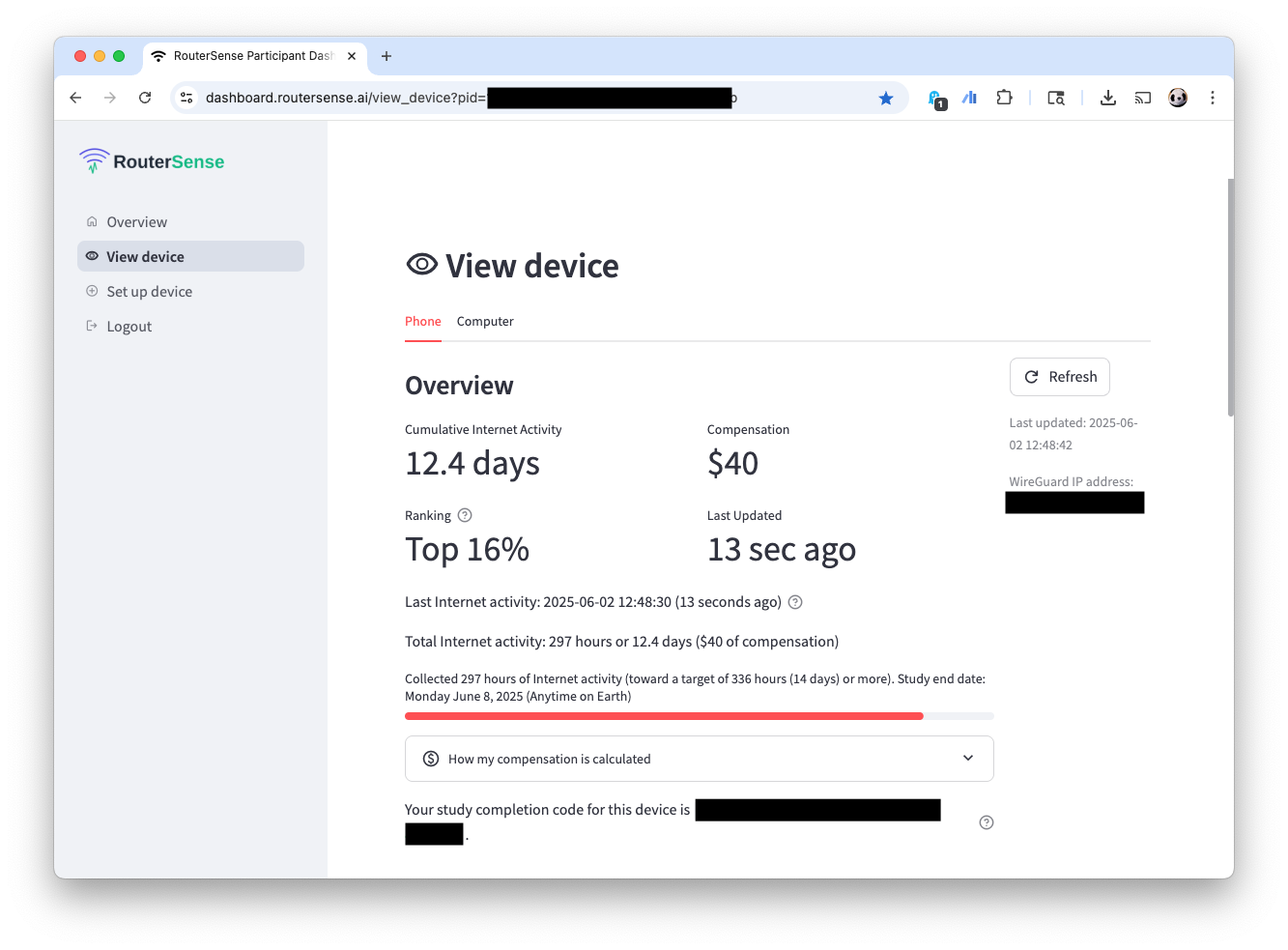


Figure D.1. Participant-facing study portal. The dashboard displays cumulative internet activity, compensation amount, and completion code, providing real-time transparency into data collection and payments.


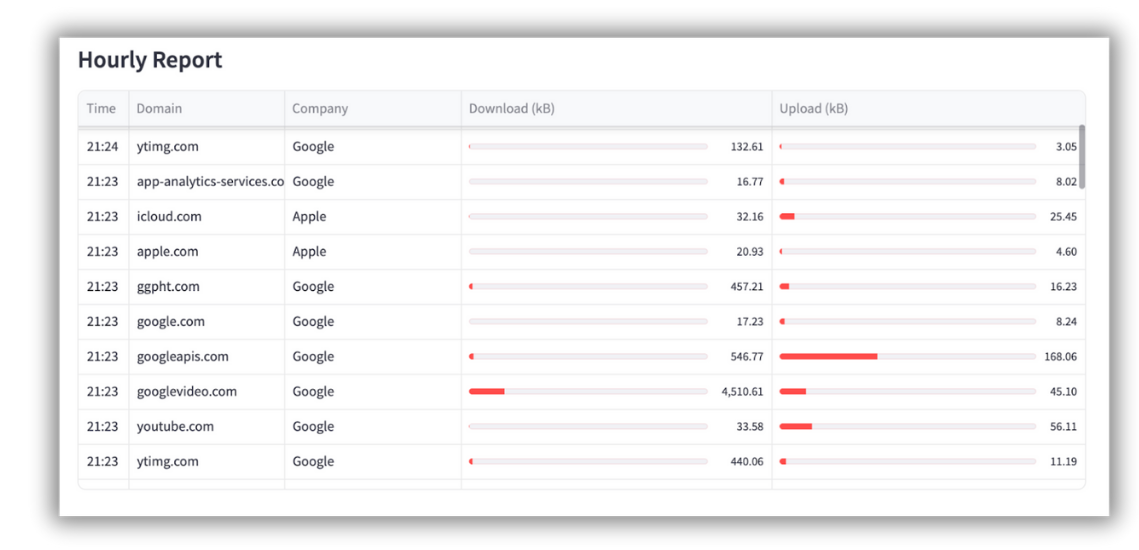


Figure D.2. Real-time table on the participant dashboard displaying hostnames contacted during app use (shown here for a YouTube session: youtube.com, googlevideo.com, ytimg.com).
