# Supplementary material for: Digital Phenotyping via Passive Network Traffic Monitoring: Prospective Observational Study in University Students
Source: JMIR Form Res. 2026 Apr 27;10:e84618. doi: 10.2196/84618 (PMC13118141; doi:10.2196/84618)
Supplement: Multimedia Appendix 6 [file formative-v10-e84618-s006.docx]

### Semi-structured Interview Questions

Q1: How’s your overall experience with the system? What was your favourite aspect of the system? What did you find the most troubling/challenging part of the system?

Q2: [If the tracking time is less than 14 days] I see that you’ve only used the system for N days. The target is 14. You’re still being paid, but any reasons why you decided not to hit the 14-day target?

Q3: [If the tracking time is greater than 14 days] I see that you’ve used the system for more than 14 days, even though we asked you to use it for 14 days. Any reasons why you decided to go beyond the 14-day target (motivation)?

Q3: Have you noticed any changes (attitudes, actions, etc) from the beginning to the end while using the system?

Q4: If unlimited time, would you have continued using the VPN? Why or why not?

Q5: Do you have any general concerns while using the VPN?

Q6: What reasons make you trust the software to keep running in the background and track your network data?

Q7: Did you continuously stay on the VPN the whole time? Did you turn it off at any point? When and Why?

Q8: How often do you check the dashboard during the study? Any difference between the beginning and end of the study?

Q9: How can we improve the user experience of the system?
