# Supplementary material for: Digital Phenotyping via Passive Network Traffic Monitoring: Prospective Observational Study in University Students
Source: JMIR Form Res. 2026 Apr 27;10:e84618. doi: 10.2196/84618 (PMC13118141; doi:10.2196/84618)
Supplement: Multimedia Appendix 7 [file formative-v10-e84618-s007.docx]

### Exit Survey Administration and Interview Procedures

Exit surveys were administered electronically following study completion. Participants were contacted via email and provided a link to the survey, which included free-text prompts for the ten most frequently used applications during the study period, as well as standardized SUS and NASA-TLX questionnaires.

Follow-up interviews were scheduled individually and conducted via Zoom. To schedule interviews and issue compensation, researchers temporarily accessed participants’ real-world identifiers (e.g., name and email address). Compensation verification was handled using a cryptographically signed completion code generated by the study portal, which encoded the payment amount without linking it to the participant’s PID. Researchers validated the authenticity of the code during the interview to confirm payment eligibility.

Interviews were transcribed using Otter AI, and researchers also took contemporaneous notes to capture qualitative observations. The complete interview guide, including all prompts and follow-up questions, is provided in Appendix E.

Optional Apple App Privacy Reports were collected only from participants who explicitly consented to share them. Participants were informed that these reports summarize app usage timing over the preceding seven days but do not reveal content accessed within apps. Shared reports were used solely as a complementary reference for app usage patterns.
