# Supplementary material for: Digital Phenotyping via Passive Network Traffic Monitoring: Prospective Observational Study in University Students
Source: JMIR Form Res. 2026 Apr 27;10:e84618. doi: 10.2196/84618 (PMC13118141; doi:10.2196/84618)
Supplement: Multimedia Appendix 8 [file formative-v10-e84618-s008.docx]

### Qualitative codebook summarizing themes, subthemes, and original codes derived from exit interview transcripts.

This table presents the full set of codes generated during thematic analysis, including higher-level themes, subthemes, and representative original codes identified through independent review and iterative axial coding. The Results section reports narrative synthesis of these codes focused on feasibility and acceptability, while this appendix provides the complete analytic structure for transparency and traceability.

| Theme | Subtheme | Original Code |
| --- | --- | --- |
| Theme 1: Security, Privacy and Risk Appraisal | Institutional and System Trust | I trust the organization/university/researcher/community/department |
|  |  | The study addresses my privacy concerns |
|  |  | I know most of the network traffic is secured |
|  |  | My concerns decreased |
|  | Active Privacy Concerns and Discomfort | Concern: I care about my data (how are you going to use it) |
|  |  | Concern: Privacy |
|  |  | Security is a concern |
|  |  | Anonymous is important |
|  |  | I don't want others to see my data/activities |
|  |  | I think others may have concerns about privacy |
|  |  | I feel uncomfortable being observed |
|  |  | I don't trust the application |
|  |  | The application/icon looks suspicious |
|  | Privacy Risk Normalization and Trade-offs | My data is being collected by other companies anyway |
|  |  | I don't really care my activities as they are not sensitive |
|  |  | My mind/thought/decisions are more important than privacy concern |
|  |  | I don't have any specific concerns |
| Theme 2: Motivations for Joining and Staying | Compensation and Gamification | Compensation is a motivation |
|  |  | The ranking is competitive |
|  |  | I use my phone more due to the ranking system |
|  |  | I don't care about the ranking anymore at the end of the study |
|  |  | The compensation rule/tier is confusing |
|  |  | VPN is free |
|  | Curiosity and Research Identity | Participant was interested in this study |
|  |  | Participants want to know more about the study |
|  |  | I want to make contribution to a study |
|  |  | I want to know more about myself |
|  | Expectations and Understanding | The study is well-explained and transparent |
|  |  | The study met my expectations |
|  |  | The study goes beyond my expectations |
|  |  | I have no expectations for this study |
|  |  | The calculation of tracking hours should be clearer |
| Theme 3: Operational Burden, Technical Frictions and Compliance | Technical Reliability and Side Effects | I face a technical issue with the system (from the developer side) |
|  |  | Turn off the VPN due to access issues |
|  |  | Turn off the VPN due to device shut down |
|  |  | VPN may interfere with other phone activities. |
|  |  | Running a VPN influences my phone battery |
|  |  | VPN slows my network speed |
|  |  | The VPN is turned off occasionally |
|  |  | I don't know why the VPN is turned off |
|  |  | I didn't realize that VPN is turned off |
|  |  | Negative emotion to figure out that VPN is not turned back on automatically |
|  |  | Negative emotion to figure out that VPN slows down the network speed |
|  |  | Negative emotion to figure out that VPN has connection issues |
|  | VPN State Management and Forgetfulness | I have to turn on the VPN after the device dead |
|  |  | I didn't turn off the VPN intentionally during the study |
|  |  | Turn off the VPN intentionally |
|  |  | I turn on and turn off the VPN frequently |
|  |  | I forgot to turn on the VPN after turning if off |
|  |  | I worried that the VPN is not turned on |
|  |  | I forgot the VPN was running in the background |
|  |  | I forgot to stop the VPN after 14 days |
|  |  | I turned off the VPN once it was more than 14 days |
|  |  | Forgetting study had ended |
|  |  | I turned off the celluar data not because off the VPN |
|  | Compliance Accuracy | Self-reported time is the same as the study period |
|  |  | Participants overestimated their tracking period in the self-report |
|  |  | Self-reported time is shorter than the study period |
|  | Understanding and Literacy | The participant is not well-knowledged about how the VPN works |
|  |  | Easy to understand for the younger generation |
| Theme 4: Behavior Change, Awareness, and Adaptation | No or Uncertain Behavior Change | I didn't change my behaviors/habits using my phone |
|  |  | I am not sure if I changed my behaviors |
|  |  | Understanding the study motivation doesn't influence my behaviors |
|  | Behavioral Increase | During the study I used my phone more |
|  |  | During the study I used my phone more but not because of the study |
|  |  | I am more aware of the time I spend on the phone |
|  |  | I was on the screen (too much) |
|  | Behavioral Reduction | It makes me want to use the phone less |
|  |  | I should use my phone less as I am being monitored |
|  |  | I use my phone less due to personal reasons |
|  | Awareness Effects | I am conscious of what I am doing on my phone |
|  |  | My sleep got worse |
|  |  | I focused more on my research/study |
|  |  | The study changed my behaviors/attitudes/routines |
|  | Avoidance and Circumvention Strategies | Turned off the VPN because I don't want to be tracked |
|  |  | I use other devices that were not tracked instead |
| Theme 5: Perceived Value, Insight and Product Feedback | Positive Value and Acceptability | Positive attitude towards using the system |
|  |  | I can benefit from using the system |
|  |  | I don't have a reason to stop using the VPN |
|  |  | Running this VPN does no harm |
|  |  | The study was fun |
|  |  | I would like to continue using the VPN for longer time |
|  | Neutral or Negative Value | Neutral attitude towards using the system |
|  |  | I gain no benefits while using the VPN |
|  |  | I am not sure if I want to use it continuously. |
|  |  | I would not like to use the system later |
|  |  | I don't have to contribute my data to a study |
|  | Usability and Unobtrusiveness | I like the device set up |
|  |  | Setting it up is easy |
|  |  | RouterSense is easy to use |
|  |  | Running the system is effortless |
|  |  | the system is seamless |
|  |  | It is unobtrusive |
|  |  | I like that the system runs in the background silently. |
|  |  | Running this VPN doesn't interfere with my life |
|  |  | I didn't find any problem with the the system |
|  | Dashboard Engagement Patterns | I sometime check the status of the system |
|  |  | I want to make sure the VPN is running by checking the dashboard |
|  |  | I have to check the vpn is running on the background |
|  |  | I check the dashboard frequently |
|  |  | I check the dashboard at the beginning of the study |
|  |  | I check the dashboard, but I don't do it frequently |
|  |  | I check the dashboard at the beginning of the study but no longer |
|  |  | I checked the dashboard more at the beginning |
|  |  | I checked the dashboard more towards the end |
|  |  | I didn't check the dashboard frequently due to personal reasons |
|  |  | I didn't compare my activities with the dashboard result |
|  |  | The participant is curious about the dashboard information |
|  | Dashboard Interpretation | The dashboard is clear |
|  |  | Dashboard is informative |
|  |  | I know my activities from the dashboard |
|  |  | My data usage matches my routines |
|  |  | The information on the dashboard alarms me |
|  | Feature Requests | Suggestion: VPN should be automatically turned on after the device is turned on |
|  |  | Suggestion: Email update for the progress |
|  |  | Suggestion: data filtering |
|  |  | Suggestion: most frequently used apps/services |
|  |  | Suggestion: network speed |
|  |  | Suggestion: hourly report |
|  |  | Suggestion: Participant ID |
|  |  | Suggestion: The dashboard should identify my activities |
|  |  | I think the UI/UX can be improved |
|  | Prior Experience and Comparison | I used other VPNs before |
|  |  | I have similar applications and tools |
|  |  | The VPN icon is a helpful sign |
|  |  | It is different from ScreenTime |
|  |  | The system has different information (periods, domains) |
