# Supplementary material for: Digital Phenotyping via Passive Network Traffic Monitoring: Prospective Observational Study in University Students
Source: JMIR Form Res. 2026 Apr 27;10:e84618. doi: 10.2196/84618 (PMC13118141; doi:10.2196/84618)
Supplement: Multimedia Appendix 9 [file formative-v10-e84618-s009.docx]

### Coverage Stratification and Aggregate Feasibility

To further contextualize coverage, bins were stratified by (a) time of day (morning: 06:00–11:59; afternoon: 12:00–17:59; evening: 18:00–23:59; night: 00:00–05:59 Eastern Time) and (b) calendar cycle (weekday vs. weekend). Participant-level coverage was summarized using mean and median coverage across individuals, while aggregate feasibility pooled bins across participants to yield overall coverage rates within each stratum.

| Scope | Feasibility (%) |
| --- | --- |
| Overall | 72.8 |
| Morning | 69.7 |
| Afternoon | 74.4 |
| Evening | 76.2 |
| Night | 70.8 |
| Weekday | 73.0 |
| Weekend | 72.1 |

Table G.1. Aggregate feasibility, calculated by pooling all monitoring bins across participants. This table reflects raw data yield, expressed as the proportion of expected bins with valid traffic.


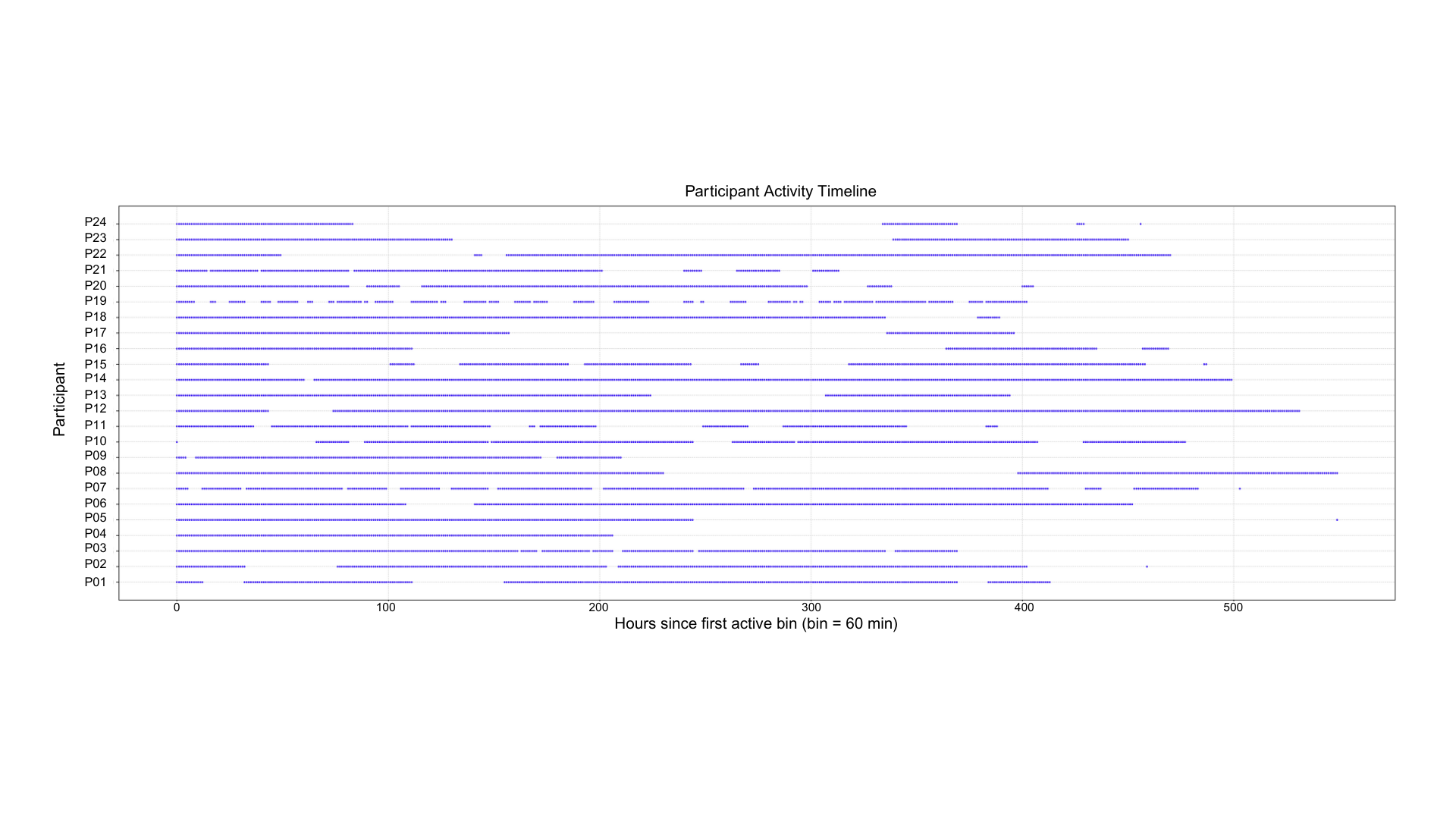


Figure G.1. Day-0–aligned VPN continuity timeline. Each dot represents a 60-minute bin with VPN traffic, normalized to participants’ first active bin. This view emphasizes individual adherence trajectories.


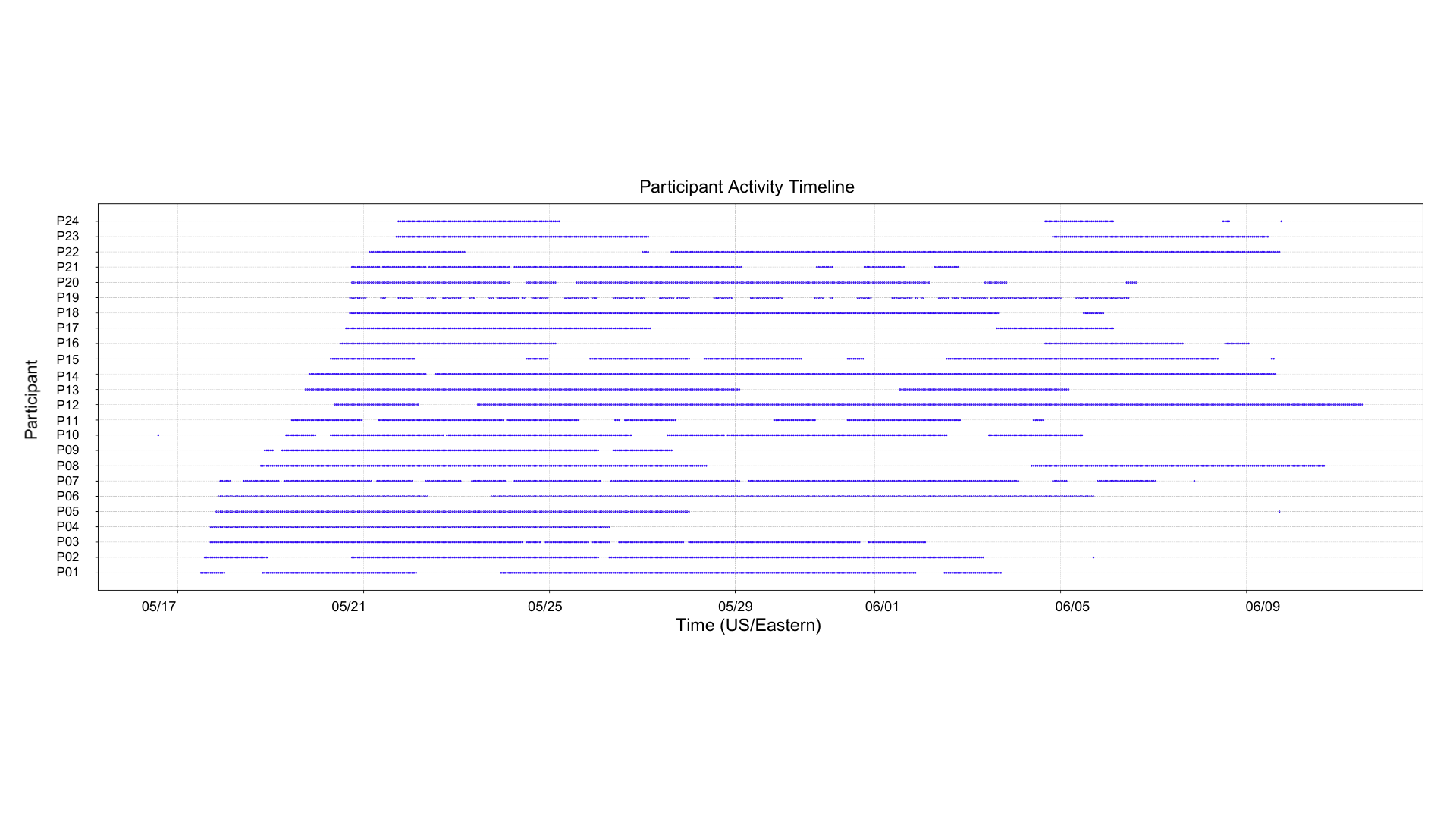


Figure G.2. Calendar-aligned VPN continuity timeline. Each dot represents a 60-minute bin with VPN traffic, ordered by enrollment date. This view emphasizes deployment feasibility.


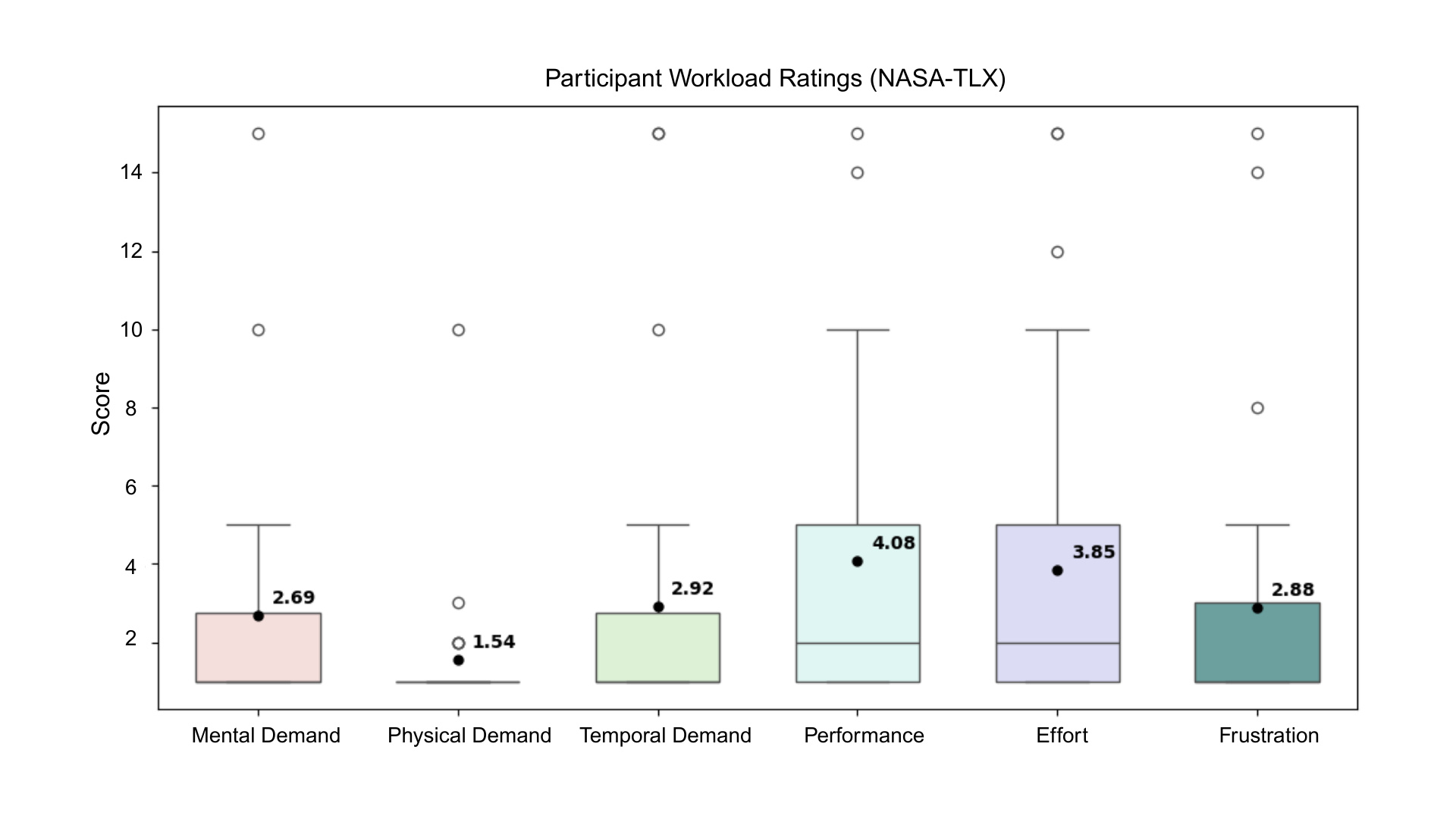


Figure G.3. Box plot of NASA-TLX scores from the exit survey (n=26), illustrating participants’ perceived workload associated with maintaining the VPN-based monitoring system in daily life. Scores are reported on a 1–21 scale (higher = greater workload).
