# Supplementary material for: Digital Phenotyping via Passive Network Traffic Monitoring: Prospective Observational Study in University Students
Source: JMIR Form Res. 2026 Apr 27;10:e84618. doi: 10.2196/84618 (PMC13118141; doi:10.2196/84618)
Supplement: Multimedia Appendix 10 [file formative-v10-e84618-s010.docx]

### App–Domain Inference Pipeline and Validation

#### Appendix I.1 Domain Normalization and Filtering

Raw hostnames recorded in App Privacy Reports (APRs) include a mixture of externally routable domains (e.g., youtube.com, api.spotify.com), local network identifiers (e.g., localhost, .local), and non-routable destinations such as IP literals. As only externally routable domains can be meaningfully associated with application backends or third-party services, hostnames were normalized and filtered prior to app-level inference.

As an initial filtering step, all non-routable or local entries—including IP literals, localhost, and .local domains—were removed. These entries reflect local network activity or device-internal communication rather than external services and therefore do not provide app-specific semantic information. This step removed 13,406 domain entries from participant-collected APRs and 12,231 domain entries from scripted APRs.

Remaining hostnames were then normalized to their registered (base) domain using public suffix rules (e.g., video.cdn.youtube.com → youtube.com) [155]. Hostnames that did not yield a valid registered domain were excluded. After normalization, participant-collected APRs contained 3,131 distinct registered domains across 29,414 valid domain entries, while scripted APRs contained 1,561 distinct registered domains across 51,078 valid domain entries.

This normalization and filtering stage operates exclusively at the domain-entry level and does not exclude applications. All 424 applications retained at least one valid registered domain following normalization. Application-level exclusion occurs only in the subsequent global app–domain disambiguation stage.

#### Appendix I.2 Global App–Domain Disambiguation

Following domain normalization, many registered domains remained associated with more than one application, reflecting shared infrastructure such as content delivery networks, analytics platforms, advertising services, and operating-system–level endpoints. To avoid ambiguous attribution, we applied a global one-to-one (1:1) app–domain disambiguation procedure over the union of participant-collected and scripted App Privacy Report (APR) data.

For each registered domain observed across the combined corpus, we enumerated the set of applications with which it appeared. Domains were retained only if they mapped to exactly one application across the entire union corpus; domains associated with multiple applications were conservatively excluded. Under this criterion, 2,563 of 3,788 registered domains (67.7%) were uniquely attributable to a single application, while 1,225 domains (32.3%) were shared across multiple applications and removed.

The impact of this disambiguation on application and domain coverage is summarized in Tables I.1 and I.2. Applying union-level disambiguation reduced the combined corpus from 424 applications to 237 applications retaining at least one uniquely attributable domain. In participant-collected APRs, 247 applications were reduced to 142, retaining 1,992 uniquely attributable domains. In scripted APRs, 288 applications were reduced to 142, retaining 661 uniquely attributable domains.

| Dataset | Apps before disambiguation | Apps after 1:1 disambiguation | Apps removed due to ambiguity |
| --- | --- | --- | --- |
| Participant (Deep-state APRs) | 247 | 142 | 105 |
| Scripted APRs | 288 | 142 | 146 |
| Union (Deep-state ∪ Scripted) | 424 | 237 | 187 |

Table I.1: App coverage across app-domain filtering stages: number of distinct applications observed at each stage of the app-domain inference pipeline. “Before disambiguation” reflects apps with at least one valid registered domain after local/IP removal and domain normalization. “After disambiguation” retains only apps with at least one domain uniquely associated with a single app across the union corpus.

| Dataset | Domains before disambiguation | Domains after 1:1 disambiguation | Domains removed as ambiguous |
| --- | --- | --- | --- |
| Participant (Deep-state APRs) | 3,131 | 1,992 | 1,139 |
| Scripted APRs | 1,561 | 661 | 900 |
| Union (Deep-state ∪ Scripted) | 3,788 | 2,563 | 1,225 |

Table I.2: Domain coverage across app–domain filtering stages: registered domains observed before and after union-level one-to-one (1:1) app–domain disambiguation. Domains shared across multiple applications in the union corpus were conservatively excluded to avoid ambiguous attribution.

To assess the practical implications of conservative disambiguation, we quantified both traffic-weighted and time-weighted attribution. Traffic-weighted attribution measured the proportion of total upload bytes associated with app-unique domains, while time-weighted attribution measured the proportion of 10-minute bins containing at least one app-attributable domain. While only 1.47% of total upload bytes were attributable to app-unique domains, 98.7% of 10-minute bins contained at least one app-attributable domain, demonstrating near-complete temporal coverage despite conservative filtering.

#### Appendix I.3 Scripted vs. Participant (“Deep-State”) Validation

To evaluate the comparability of app-identifying domains recovered from brief scripted app interactions and those observed during prolonged real-world (“deep-state”) use, we compared domains observed in scripted and participant-collected App Privacy Reports (APRs) after union-level app–domain disambiguation. As meaningful comparison requires overlap in application coverage, the analysis was restricted to the 47 applications present in both datasets that retained at least one uniquely attributable registered domain.

To assess semantic identifiability, an application was considered identifiable if it exhibited at least one domain clearly and unambiguously referring to the app’s name, brand, or core service (e.g., youtube.com for the YouTube app). When this rule-based criterion was insufficient, two authors independently evaluated remaining domains in the context of the app’s name and primary functionality, with disagreements resolved through discussion to reach consensus.

Within the shared cohort, 30 of the 47 applications were semantically identifiable based on domains observed during brief scripted launches. Participant-collected (“deep-state”) usage enabled semantic identification for 31 applications, including all 30 applications identified via scripted interactions plus one additional application. While sustained or authenticated usage introduced additional domains for some applications, these domains provided limited incremental benefit for app identification and more often reflected shared infrastructure or generic services rather than app-specific signals.

Table I.3 summarizes the overlap in application coverage between participant-collected (“deep-state”) and scripted APRs after union-level app–domain disambiguation. As meaningful comparison requires overlap in application coverage, the analysis was restricted to the 47 applications present in both datasets, each retaining at least one uniquely attributable registered domain under the union-level one-to-one (1:1) filtering procedure.

| Metric | Value |
| --- | --- |
| Apps retained in both scripted and deep-state APRs | 47 |
| Apps retained only in deep-state APRs | 95 |
| Apps retained only in scripted APRs | 95 |

Table I.3: Overlap in app coverage between participant-collected (“deep-state”) and scripted APRs after union-level disambiguation. The substantial overlap indicates that brief scripted interactions recover app-identifying domains for many apps also observed during naturalistic use.
